# Food Pricing Strategies, Population Diets, and Non-Communicable Disease: A Systematic Review of Simulation Studies

**DOI:** 10.1371/journal.pmed.1001353

**Published:** 2012-12-11

**Authors:** Helen Eyles, Cliona Ni Mhurchu, Nhung Nghiem, Tony Blakely

**Affiliations:** 1 National Institute for Health Innovation, University of Auckland, Auckland, New Zealand; 2 Department of Public Health, University of Otago, Wellington, New Zealand; University of Cambridge, United Kingdom

## Abstract

**Background:**

Food pricing strategies have been proposed to encourage healthy eating habits, which may in turn help stem global increases in non-communicable diseases. This systematic review of simulation studies investigates the estimated association between food pricing strategies and changes in food purchases or intakes (consumption) (objective 1); Health and disease outcomes (objective 2), and whether there are any differences in these outcomes by socio-economic group (objective 3).

**Methods and Findings:**

Electronic databases, Internet search engines, and bibliographies of included studies were searched for articles published in English between 1 January 1990 and 24 October 2011 for countries in the Organisation for Economic Co-operation and Development. Where ≥3 studies examined the same pricing strategy and consumption (purchases or intake) or health outcome, results were pooled, and a mean own-price elasticity (own-PE) estimated (the own-PE represents the change in demand with a 1% change in price of that good). Objective 1: pooled estimates were possible for the following: (1) taxes on carbonated soft drinks: own-PE (*n = *4 studies), −0.93 (range, −0.06, −2.43), and a modelled −0.02% (−0.01%, −0.04%) reduction in energy (calorie) intake for each 1% price increase (*n = *3 studies); (2) taxes on saturated fat: −0.02% (−0.01%, −0.04%) reduction in energy intake from saturated fat per 1% price increase (*n = *5 studies); and (3) subsidies on fruits and vegetables: own-PE (*n = *3 studies), −0.35 (−0.21, −0.77). Objectives 2 and 3: variability of food pricing strategies and outcomes prevented pooled analyses, although higher quality studies suggested unintended compensatory purchasing that could result in overall effects being counter to health. Eleven of 14 studies evaluating lower socio-economic groups estimated that food pricing strategies would be associated with pro-health outcomes. Food pricing strategies also have the potential to reduce disparities.

**Conclusions:**

Based on modelling studies, taxes on carbonated drinks and saturated fat and subsidies on fruits and vegetables would be associated with beneficial dietary change, with the potential for improved health. Additional research into possible compensatory purchasing and population health outcomes is needed.

*Please see later in the article for the Editors' Summary*

## Abstract 2

A systematic review of simulation studies conducted by Helen Eyles and colleagues examines the association between food pricing strategies and food consumption and health and disease outcomes.

## Editors' Summary

**Background:**

For the first time in human history, non-communicable diseases (NCDs) are killing more people than infectious diseases. Every year, more than 35 million people die from NCDs—nearly two-thirds of the world's annual deaths. More than 80% of these deaths are in developing countries, where a third of NCD-related deaths occur in people younger than 60 years old. And NCDs are not just a growing global public health emergency. They are also financially costly because they reduce productivity and increase calls on health care systems worldwide. Cardiovascular diseases (conditions that affect the heart and circulation such as heart attacks and stroke), cancers, diabetes, and chronic respiratory diseases (long-term diseases that affect the lungs and airways) are responsible for most NCD-related illnesses and death. The main behavioral risk factors for all these diseases are tobacco use, harmful use of alcohol, physical inactivity, and unhealthy diets (diets that have a low fruit and vegetable intake and high saturated fat and salt intakes).

**Why Was This Study Done?:**

Improvements in population diets and reductions in salt intake are crucial for the control and prevention of NCDs, but how can these behavioral changes be encouraged? One potential but poorly studied strategy is food pricing—the introduction of taxes on unhealthy foods (for example, foods containing high levels of saturated fat) and subsidies on healthy foods (for example, foods high in fiber). However, although a tax on soft drinks, for example, might decrease purchases of these high-sugar drinks, it might also increase purchases of fruit juices, which contain just as much sugar and energy as soft drinks (“compensatory purchasing”), and thus undermine the intended health impact of the tax. Because randomized controlled trials of the effects of food pricing strategies are difficult to undertake, many researchers have turned to mathematical models (sets of equations that quantify relationships between interventions and outcomes) to provide the evidence needed to inform policy decisions on food taxes and subsidies. In this systematic review (a study that uses predefined criteria to identify all the research on a given topic), Helen Eyles and colleagues investigate the association between food pricing strategies and food consumption and NCDs by analyzing the results of published mathematical modeling studies of food pricing interventions.

**What Did the Researchers Do and Find?:**

The researchers identified 32 studies that met their predefined inclusion criteria, which included publication by researchers in a member country of the Organisation for Economic Co-operation and Development (a group of largely developed countries that promotes global development). Most of the studies were of low to moderate quality and provided uncertain and varying estimates of the impact of pricing on food consumption. Where three or more studies examined the same pricing strategy and consumption or health outcome, the researchers calculated the average change in demand for a food in response to changes in its price (“own-price elasticity”). For taxes on carbonated soft drinks, the average own-price elasticity was −0.93; that is, the models predicted that a 1% increase in the price of soft drinks would decrease consumption by 0.93%. The modeled reduction in the proportion of energy intake from saturated fat resulting from a 1% increase in the price of saturated fats was 0.02%. Finally, although the researchers' analysis suggested that for each 1% reduction in the price of fruits and vegetables, consumption would increase by 0.35%, they also found evidence that such a subsidy might result in compensatory purchasing, such as a reduction in fish purchases.

**What Do These Findings Mean?:**

These findings suggest that pricing strategies have the potential to produce improvements in population diets, at least in developed countries, but also highlight the need for more research in this area. Notably, the researchers found insufficient data to allow them to quantify the effects of pricing strategies on health or to analyze whether the effect of pricing strategies is likely vary between socio-economic groups. Given their findings, the researchers suggest that future modeling studies should include better assessments of the unintended effects of compensatory purchasing and should examine the potential impact of food pricing strategies on long-term health and NCD-related deaths. Finally, they suggest that robust evaluations should be built into the implementation of food pricing policies to answer some of the outstanding questions about this potential strategy for reducing the global burden of NCDs.

**Additional Information:**

Please access these websites via the online version of this summary at http://dx.doi.org/10.1371/journal.pmed.1001353.

- The US Centers for Disease Control and Prevention provides information on all aspects of healthy living, on chronic diseases and health promotion, and on non-communicable diseases around the world
- The Global Noncommunicable Disease Network (NCDnet) aims to help low- and middle-income countries reduce NCD-related illnesses and death through implementation of the 2008-2013 Action Plan for the Global Strategy for the Prevention and Control of Noncommunicable Diseases (also available in French); NCDnet's Face to face with chronic disease webpage is a selection of personal stories from around the world about dealing with NCDs
- The American Heart Association and the American Cancer Society provide information on many important risk factors for non-communicable diseases and include some personal stories about keeping healthy

## Introduction

Non-communicable diseases (NCDs) including heart disease, stroke, diabetes, cancers, and chronic respiratory disease are the leading preventable causes of global morbidity and mortality [1]. Furthermore, these diseases place substantial burden on national economies, contribute to poverty, and are a major cause of health inequalities [2]–[4]. Nutrition-related risk factors such as low fruit and vegetable intake and high saturated fat and sodium intakes are causative risk factors for NCDs. Improving population diets and reducing sodium intake were recently identified as two priority areas for action at the United Nations High Level Meeting on Prevention and Control of NCDs in September 2011 [5]. If these changes in population diet take place, the interventions will support the global goal of reducing NCD death rates and averting tens of millions of premature deaths within the next decade [3].

Price is one of the most important factors influencing food choice [6], and pricing strategies (food taxes and subsidies) have been proposed as a means to improve population diets and reduce rates of obesity and NCDs. Food taxes are also of interest to national and state governments because of their revenue-raising potential. Such policies have been used successfully in other areas of public health [7], the best example of which is tobacco smoking, where price increases in cigarettes led to immediate and permanent decreases in sales and in the global prevalence of smoking [8],[9]. Nonetheless, despite differential application of goods and services tax or value added tax to food in some countries [10], the application of such taxes is largely driven by revenue-raising imperatives, and food pricing policy with the aim of improving population diets is a relatively untested concept.

In 2008 the World Health Organization recommended that policy be used to influence food prices “in ways that encourage healthy eating” [11]. Economic theory suggests that increasing the price of foods high in nutrients counter to health (such as excess energy [caloric content], sodium, sugar, and saturated fat) and/or reducing the price of foods high in nutrients that are pro-health (such as fibre and unsaturated fat) may improve the nutritional quality of diets consumed, raise revenue to support other population health interventions or government expenditures, and send a clear message to consumers about which foods are healthier [12].

The effects of altering the price of food on purchase volumes are estimated using price elasticities (PEs). Own-PEs refer to changes in the demand of a good/food due to changes in its own price; cross-PEs refer to changes in the demand of a good/food in response to price changes in another related good/food. For example, a price increase in butter is likely to produce a reduction in butter purchases (own-PE), but also displacement or compensatory purchasing of margarine as a substitute (cross-PE). The higher the PE, the more purchase volume changes as a result of price changes. The magnitude of a cross-PE depends on whether a non-targeted good/food is a suitable substitute for the good/food targeted for a tax or subsidy. However, sometimes unintended compensatory or displacement purchasing may occur and undermine the health objective of the tax or subsidy. For example, individuals faced with a subsidy on fruits and vegetables may purchase more fruits and vegetables (own-PE), but also purchase more foods high in saturated fat and sodium (cross-PE).

The possibility of compensatory purchasing is one concern with food taxes and subsidies. Another is that taxes may be regressive by disproportionately affecting lower income households [12], if lower income households spend a greater percentage of their household budget on food items that are taxed (or less on food items that are subsidised), even allowing for changes in purchasing behaviour due to the pricing intervention. Nonetheless, a higher PE (both own and cross) among lower income households could mitigate such concerns, and even help reduce health disparities [13].

Despite increasing interest, evidence of the effect of food pricing strategies on consumption, health, and disease is limited. Pricing interventions are difficult to evaluate using traditional robust research methodologies, such as randomised controlled trials (RCTs), because pricing strategies are difficult to undertake in the real world setting and RCTs are generally too short to determine long-term health effects. Nonetheless, two such pragmatic RCTs have been undertaken (one in the United States [14] and one in New Zealand [15]), and three are about to start (two in Australia [16] [J. Brimblecombe, personal communication] and one in the Netherlands [17]). Both of the completed trials found that subsidies on fruits and vegetables increased fruit and vegetable purchases (one of the studies evaluated effects on purchases of other food groups: no significant differences were found).

The best evidence for a change in the price of food improving health and disease would be provided by RCTs, with factors measured with accuracy and precision across multiple heterogeneous populations. However, as this is an unrealistic expectation, evidence may be sought from simulation models, which help to bridge the gap between empirical research and long-term health outcomes [18]. Simulation modelling involves the development of causal diagrams and policy-oriented computer models that use a collection of mathematical equations to quantify the relationships between interventions and particular outcomes of interest [18],[19]. Such models simplify the complex ecological system surrounding an intervention to a few quantifiable possibilities.

Figure 2 shows a causal diagram illustrating the relationships between food price and health or disease. Four steps, or types of variables, are included: food price, food intake, nutrient and energy intake, and the effect on health or disease. These steps are linked together (the “model structure”) by assumptions, about which there is some uncertainty. “Parameters,” or “inputs,” such as PEs and nutrient conversion tables (about which there is also some uncertainty), are entered into the model. Mathematical predictions of how the change in one or more variables influences another or multiple variables in the next step, in this case, for example, relative risks, are referred to as link functions. Importantly, population heterogeneity can alter the magnitude of these parameters. For example, dietary norms and tastes can alter PE values across populations and sub-populations. For wealthy populations for which food is a small proportion of total expenditure, food intake may not be particularly sensitive to food price. Cultural norms about diets also mean some foods are considered “staples” and may not alter in purchase volumes with price. In this case, such foods are relatively inelastic.

One systematic review of simulation studies exploring the association of food price with consumption, health, and disease has been undertaken to date (*n = *16 studies) [20]. This previous review concluded that food taxes and subsidies have the potential to influence food consumption and health considerably, particularly when such taxes/subsidies are large (∼15% of product price or more) [20]. Other reviews of pricing strategies have reached similar conclusions [19],[21]–[24]. However, these and the previous systematic review provided narrative summaries, and although such summaries are useful, they are not able to provide policy makers with a clear message regarding the likely magnitude and direction of effects on food consumption and health. Moreover, reviews to date have not comprehensively addressed issues of quality in the PEs or epidemiological models, or specifically focused on the impacts of pricing strategies for different socio-economic groups. Furthermore, because of the recent rise in global food prices and increasing interest in food pricing strategies, a number of simulation modelling studies have been undertaken in recent years that were not included in the previous systematic review (the current review identifies 15 additional studies). Therefore, a comprehensive, updated systematic review of simulation studies is timely and important.

The overall aim of this systematic review was to assess and quantitatively evaluate (where possible) the potential of food taxes and subsidies to improve population diet (intake or purchases), improve health (risk factors such as cholesterol and blood pressure), and reduce NCDs in member countries of the Organisation for Economic Co-operation and Development (OECD), as estimated with simulation models. Specific objectives were to assess: (1) the magnitude of the association of food taxes and subsidies with changes in food and nutrient intake, (2) the magnitude of the association of taxes and subsidies with health and disease states, and (3) the evidence for heterogeneity in these findings by socio-economic position. Objective 1 is driven largely by PE estimates used in the simulation model, and objective 2 is additionally driven by the epidemiological component. Therefore, quality assessments of these two components of the simulation models were included. In addition to providing, to our knowledge, the first pooled estimates of the association of food pricing strategies with consumption, health, and NCDs, we recommend better designs, assessments of quality, and improved reporting of future simulation studies.

## Methods

The methods used to systematically collect studies for this review were based on those outlined in the *Cochrane Handbook for Systematic Reviews of Interventions* [25]. However, meta-analysis techniques were not used in combining the findings of simulation studies, as such models use a variety of structures and mathematical techniques to estimate the impact of an intervention, in contrast to, for example, RCTs, which are amenable to meta-analysis because they follow one relatively standard technique or method. Nonetheless, where possible, estimates of PEs and their immediate impacts on consumption (from epidemiological models) were synthesised as one important aspect of results. Further details are provided in “Data Extraction and Synthesis” below. A protocol for this review has not been published separately. The PRISMA checklist is provided as Text S1.

### Selection of Studies

Simulation modelling studies published in peer-reviewed journals or as scientific reports were included. The following definition was used for defining eligible simulation models to include in this review: a study that uses a collection of mathematical equations to quantitatively map the relationships between food price change and resultant change in at least one of the following: food consumption (intake or food purchases), health status (biological risk factors such as blood pressure and cholesterol), and NCDs (modified from [19]). This review focused on the potential of food pricing strategies to improve the *quality* of population diets and associated health and NCD outcomes. As such, the outcomes of interest were largely associated with the consequences of over- rather than under-nutrition. For example, health outcomes included blood cholesterol and blood pressure, and NCD outcomes included obesity, cardiovascular disease (CVD), and cancer. To exclude studies where under-nutrition was the primary driver of food pricing strategies, the review was limited to studies undertaken in one of the 34 member countries of the OECD [26]. As explained above, RCTs and observational studies were not included, although such studies will have contributed substantively to the development of the simulation models.

### Search Strategy and Data Sources

Major electronic databases (Medline, Embase, and Food Science and Technology Abstracts) were searched for relevant journal articles published between 1 January 1990 and 24 October 2011. The year 1990 was chosen to ensure included studies were relevant to current dietary habits and practices and mathematical models. The search strategy used for Medline is presented below; this search strategy was modified accordingly for other databases. Searches were limited to articles published in English. Relevant articles and grey literature, including economic literature, technical reports, and working papers, were also identified through Google Web and Google Scholar, and by searching the bibliographies of included studies. Enquiries were also made to key experts in the field. One author (H. E.) assessed all abstracts for suitability for inclusion; a second author (C. N. M.) was available to address any queries.

#### Search strategy for Medline

The following search algorithm was used: (1) exp Food/, (2) food$.mp, (3) exp Diet/, (4) nutrition$.mp., (5) exp Eating/, (6) exp Food Habits/, (7) food purchas$.mp., (8) 1 or 2 or 3 or 4 or 5 or 6 or 7, (9) price$.mp., (10) exp Nutrition Policy/or exp Health Policy/, (11) subsid$.mp., (12) discount$.mp., (13) cost$.mp., (14) taxes/or tax exemption/, (15) exp “Cost Control”/, (16) pricing strateg$.mp., (17) 9 or 10 or 11 or 12 or 13 or 14 or 15 or 16, (18) exp Computer Simulation/, (19) simulation model$.mp., (20) exp Systems Analysis/, (21) systems model$.mp., (22) micro simulation$.mp., (23) macro simulation$.mp., (24) simulat$.mp, (25) 18 or 19 or 20 or 21 or 22 or 23 or 24, (26) 8 and 17 and 25, (27) limit 26 to (English language and year = “1990–Current”).

### Data Extraction and Synthesis

The following data were extracted for all included studies: first author, year, country, food groups included in the simulation model, interventions modelled, datasets used, inclusion of lower socio-economic groups, outcomes, impact, quality, and feasibility issues addressed (definition of healthy/less healthy foods, and format and magnitude of tax/subsidy). Assessment of the quality of included studies focussed on two components. The first component addressed the PE estimates, in particular, whether both own- and cross-PEs were calculated/sourced within a complete demand system (i.e., including all food groups), whether PEs were developed using long-run input data (definition provided below), whether appropriate PE input data were used (relevant country), and whether uncertainty of PE was addressed (i.e., through reporting of error or uncertainty). Where relevant, the application of differential PEs for socio-economic sub-groups was also recorded. The second component addressed the epidemiological model components or parameters/inputs and link functions used in the simulation, in particular, whether the study used valid and appropriate consumption data for the country of interest, whether the study attempted to validate or calibrate the model, and whether parametric uncertainty or variation of the model inputs was addressed (e.g., through sensitivity analyses). It is important to note, though, that no attempt was made to undertake a thorough assessment of the structural uncertainty of the models, including the selection of relative risk functions.

Further description of how these quality dimensions were defined and assessed, including the types of inputs used for the models, is provided in Text S2.

#### Assessment of studies as high quality

High-quality studies were defined as those meeting all of the criteria outlined above. However, no study included in the review met all quality criteria or was defined as high quality. Therefore, studies meeting the following four key criteria were considered “moderately high quality”: (1) own- and cross-PEs calculated from a complete food demand system including at least eight food groups and 70% of all possible food categories (models were still considered complete if only missing restaurant and/or take-away foods); (2) long-run input data with sufficient variation in price used to estimate PEs, i.e., data collected continuously across the survey population, or at least monthly for at least 2 y; (3) own country consumption, prevalence, and mortality data used to populate epidemiological model; (4) large sample (population-based; *n*≥1,000).

Within objectives 1 and 2, studies were disaggregated by type of pricing policy (tax, subsidy, or combination). Where there were three or more studies within each major category that were sufficiently alike in terms of pricing strategy and outcome, findings were quantitatively pooled to produce a mean PE estimate. Pooled estimates were calculated in two ways, as studies may have assessed more than one tax or subsidy rate. First, all unique tax/subsidy rates and corresponding impacts on consumption were included, thus studies could contribute more than one value. Second, in order to test the effect of correlation of values from the same study, we created a summary mean for each study from the contributing values for that study, and the robustness of the first analysis was assessed. A tax pass-through rate of one (that is, a one-dollar change in tax leads to a dollar change in food price, in the same direction) was assumed. Findings from all other studies were combined in narrative summary.

### Ethics Statement

An ethics review was not required for this work.

## Results

**Figure 1:**
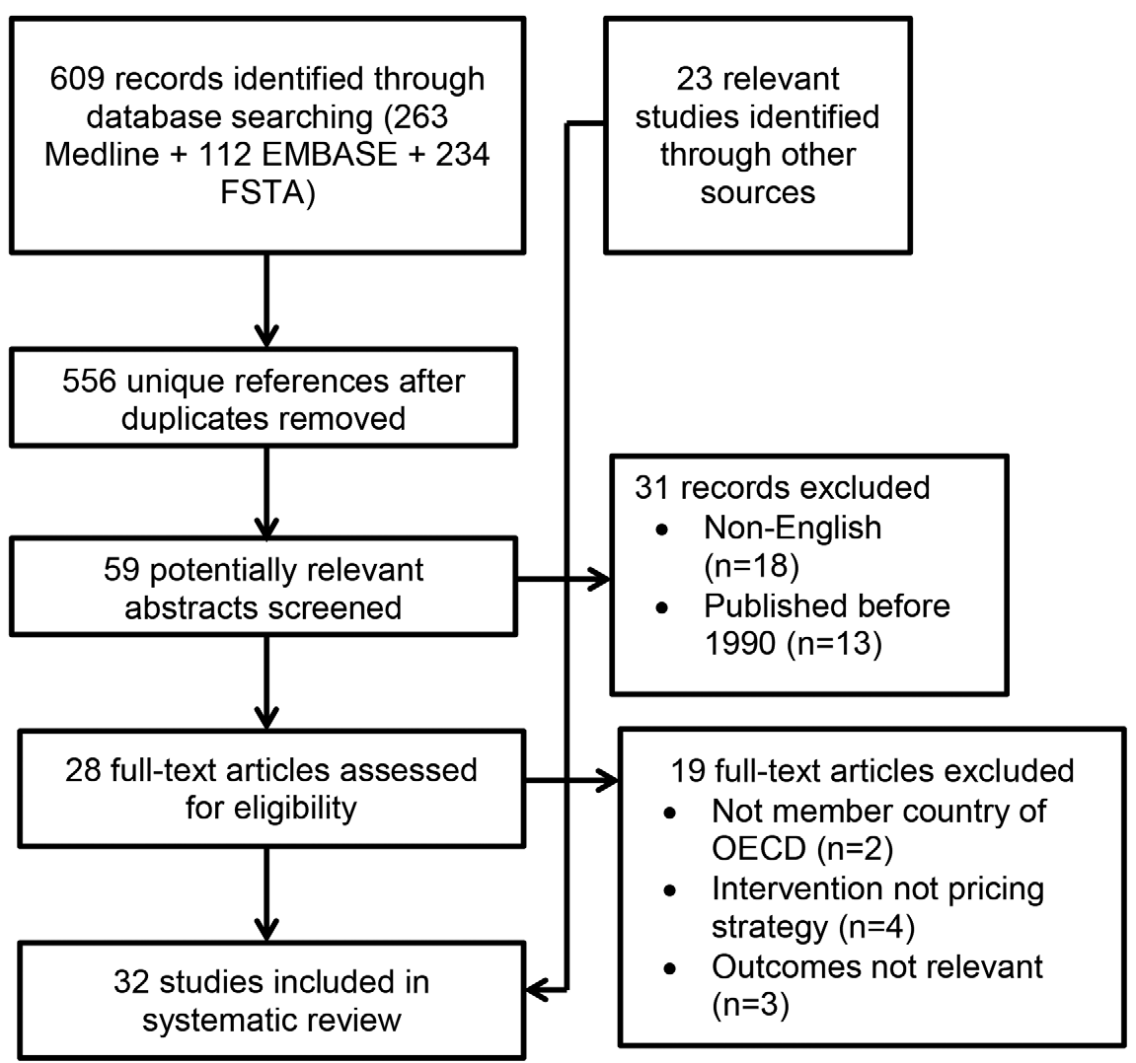
Flow diagram for study selection. FSTA, Food Science and Technology Abstracts.

The study selection process is shown in Figure 1. The structured literature search identified 556 potentially relevant unique citations. Of these, 59 potentially relevant abstracts were screened: 31 records were excluded; 18 were published in languages other than English, and the remaining 13 were published prior to 1990. The titles of the non-English articles were available for review in English and were thus reviewed for suitability; all 18 were judged to be outside the scope of the review. Twenty-eight full-text papers were screened for eligibility, of which nine met inclusion criteria for the review. Figure 1 lists reasons for exclusion of the remaining 19 manuscripts. Two articles were excluded because they were from non-OECD countries (both were from Egypt [27],[28]). The first evaluated the effect of the Egyptian food subsidy programme on mother's BMI status. This programme provides all households with a price reduction on bread and flour (57%), and low-income households with price reductions on sugar and cooking oil (43% to 62%, depending on income level) [27]. Seven-day diet recall data from the 1997 Egyptian Integrated Household Survey (*n = *2,000 rural and urban households) were linked with pricing information to determine own- and cross-PEs for energy-dense and energy-dilute food. Mother's BMI (from the 1997 Egyptian Integrated Household Survey) was found to be positively associated with lower priced, energy-dense food. However, there was no association between income and BMI. The second study evaluated a reform of the subsidy programme, but did not include any of the outcomes specified for inclusion in this review. Eighteen additional eligible papers and reports were identified through Google Web, Google Scholar, and the bibliographies of included studies. Five further eligible papers and reports were identified through key experts. Thirty-two studies (19 peer-reviewed papers and 13 other types of reports) met all inclusion/exclusion criteria and were included in the review.

**Figure 2:**
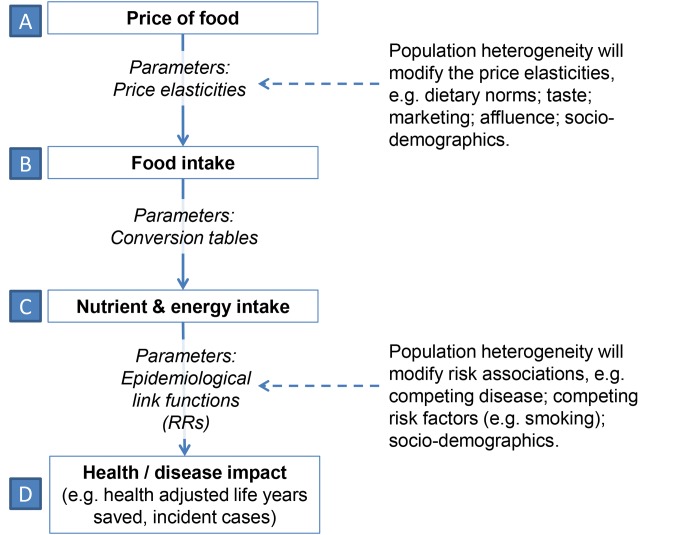
Causal diagram for simulation model illustrating how food price leads to health or disease impact. Four steps, or types of variables: food price (A), food intake (B), nutrient and energy intake (C), and the impact on health or disease (D). RRs, relative risks.

**Figure 3:**
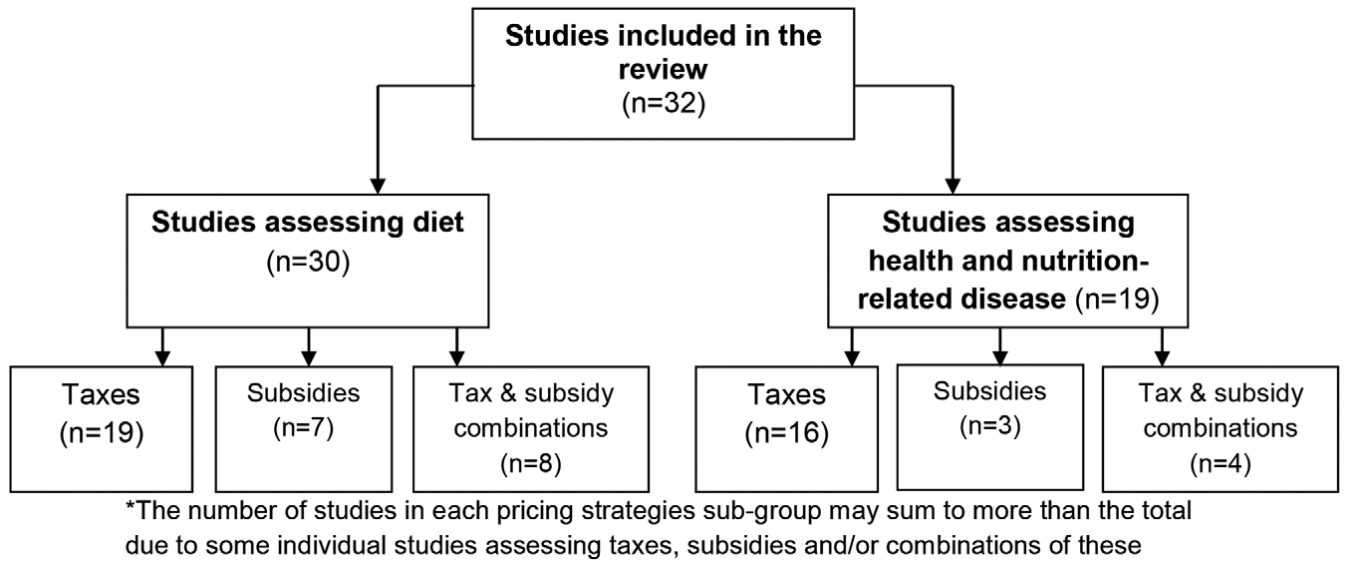
Summary of included studies and presentation of findings of the review*.

Figure 3 illustrates the number of studies included in each sub-category for presentation of the review findings (note that some studies have been included in more than one sub-category).

### Characteristics of Included Studies

A summary of included studies follows. For a detailed description of each study, please refer to Text S2.

#### Studies assessing diet (food/nutrient intake or purchases)

Thirty simulation modelling studies assessed the impact of food pricing on diet outcomes (i.e., food/nutrient intake or purchases): 17 assessed food taxes [13],[29]–[45], six assessed subsidies [33],[39],[42],[45]–[47], and seven assessed combinations of food taxes and subsidies [8],[39],[40],[42],[45],[48]–[51] (note that six of the 30 individual studies assessed taxes, subsidies, and/or combinations of taxes and subsidies and as such have been included in more than one category above [33],[39],[40],[42],[45],[51]). Thirty-six food and 12 nutrient outcomes were assessed by the 30 studies included in this section of the review.

#### Studies assessing health and disease outcomes

Nineteen simulation modelling studies assessed the impact of food pricing on health and disease outcomes: 15 assessed taxes [31],[34]–[38],[40],[41],[43],[45],[52]–[56], three assessed subsidies [45],[56],[57], and four assessed combinations of food taxes and subsidies [40],[45],[50],[52] (note that four of the 19 individual studies assessed taxes, subsidies, and/or combinations of taxes and subsidies and as such have been included in more than one category above [40],[45],[52],[56]. Six health and 16 disease outcomes were assessed by the 19 studies included in this section of the review.

### Quality of Included Studies

A table summarising the quality of studies, including the inputs used for the models, is included in Text S2. For the PE quality component, 15/32 studies used a complete demand system, 24/32 studies used what should be long-run PEs, 22/32 included own- and cross-PEs, 11/32 used valid and appropriate source data for PEs, and one study addressed the uncertainty of PE values with probabilistic sensitivity analyses. For the epidemiological component, 30/32 studies used valid and appropriate source data, 25/32 used consumption data that was collected/projected over time, 7/32 undertook some type of sensitivity analyses, and no studies validated or calibrated their model (Table 1).

**Table 1:** Summary of simulation modelling studies assessing the impact of taxes on food/nutrient consumption (*n = *19 studies).

Taxed Food or Nutrient	Measured Food or Nutrient	Impact of Tax			Mean (Standard Deviation) Own-PE^a^
		Number of Studies Reporting Increased Consumption	Number of Studies Reporting No Impact	Number of Studies Reporting Decreased Consumption	
**All sweetened beverages**	Diet sugar-sweetened beverages		1 [30]		
	Fruit drinks			2 [30],[44]	
	Sports drinks			2 [30],[44]	
	Regular ready-to-drink teas			1 [30]	
	Diet ready-to-drink teas		1 [30]		
	Flavoured water			1 [30]	
	Energy drinks			2 [30],[44]	
	Coffee	1 [44]		1 [30]	
	Carbonated sugar-sweetened beverages			2 [30],[44]	
	Whole milk			1 [44]	
	Bottled water			1 [44]	
	Sugar			1 [44]	
	Low-fat milk	1 [44]			
	Fruit juice	1 [44]			
	Tea	1 [44]			
	All sweetened beverages			1 [8]	
	Sugar-sweetened beverages			1 [8]	
**Carbonated soft drinks**	*Carbonated soft drinks*			*4 * [32],[33],[37],[58]	*−0.93 (0.91)*
	Sports drinks			2 [32],[37]	
	Snack foods			1 [58]	
	Fruit drinks			1 [37]	
	Whole milk			1 [37]	
	Low-fat milk		1 [37]		
	Fruit juice		1 [37]		
	Bottled water	1 [37]			
	Coffee		1 [37]		
	Tea		1 [37]		
	*Energy*			[13],[37],[38]	
**Saturated fat**	Whole milk	**1 [39]**		1 [35]	
	Cheese	**1 [39]**		1 [35]	
	Butter	**1 [39]**		1 [35]	
	Biscuits			1 [35]	
	Buns, cakes, and pastries			1 [35]	
	Puddings and ice cream			1 [35]	
	*Saturated fat*			***5*** **[35],[36],[39],[40],[42]**	
	Sodium	**1 [36]**			
	Non-milk extrinsic sugar			**1 [36]**	
	Energy	**1 [36]**		**1 [40]**	
	Fruits and vegetables	**1 [40]**		**2 [36],[39]**	
	Total fat	**1 [39]**			
	Salt	**1 [40]**			
	Sugar	**1 [42]**			
	Fibre	**1 [42]**			
**Sugar**	Bread			1 [45]	
	Meat	1 [45]			
	Fish		1 [45]		
	Fruits and vegetables		1 [45]		
	Sugar and sweets			1 [45]	
**Less healthy/junk foods**	Saturated fat	**1 [36]**			
	Salt			**1 [36],[40]**	
	Non-milk extrinsic sugar			**1 [36]**	
	Energy			**1 [36],[40]**	
	Fruits and vegetables			**1 [36],[40]**	
	Saturated fat			**1 [40]**	
	Less healthy/junk foods			1 [41]	

Bold indicates studies that were considered moderately high quality (see Text S2). Italicised rows represent tax scenarios where sufficient studies were available to quantitatively aggregate findings (≥3 similar studies).

a Mean PE value calculated only where ≥3 studies per major category.

Based on our conservative quality criteria, only seven studies were considered “moderately high quality” [29],[36],[39],[40],[42],[52],[55]. Three of these studies were essentially evolutionary studies undertaken by the same/similar group of researchers [36],[40],[52].

A summary of the overall findings of the review is provided in Box 1.

**Box 1. Summary of Key Findings**

- **Impact on food and nutrient consumption (objective 1):** Quantitative summary was possible for three scenarios, all of which suggested impacts on consumption would be pro-health.
  1. The own-PE for carbonated soft drinks was −0.93 (range, −0.06, −2.43), and the resulting modelled reduction in energy consumption was −0.02% (range, −0.01%, −0.04%) for each 1% increase in price.
  2. The modelled reduction in saturated fat resulting from a saturated fat tax was −0.02% (range, −0.01%, −0.04%) of total energy for each 1% increase in price.
  3. The own-PE for a subsidies on fruits and vegetables was −0.35 (range, −0.21, −0.77).
- **Impact on health and disease (objective 2) and differences by socio-economic group (objective 3):** Variability of food taxes and subsidies and types of consumption, health, and disease outcomes assessed prevented any pooled analyses. However, a few conclusions are possible.
  1. Higher quality studies estimated that dairy/saturated fat taxes may increase mortality from CVD and CHD (*n* = 1 study), and less healthy/junk food taxes may increase overall mortality (*n* = 1 study) and mortality from stroke and CVD (*n* = 2 studies).
  2. Most (11/14) studies assessing absolute impacts for lower socio-economic groups estimated that effects on food and nutrient consumption, and health and disease, would be pro-health. Relative impacts may also be greater for lower income groups, and thus food taxes and subsidies have the potential to be pro-equity.
- **Other key findings:**
  1. The majority of included studies (25/32) were of low quality. Furthermore, there was substantial variability in model structures, data inputs, and the types and magnitudes of food taxes and subsidies assessed.
  2. There was also some evidence that pricing strategies may result in unintended compensatory buying through cross-PEs; two moderately high quality studies estimated a potential increase in consumption of sodium in response to a saturated fat tax, and a potential increase in mortality from CVD in response to a tax on less healthy foods.

### Objective 1: Estimated Impact on Food and Nutrient Consumption

#### Taxes

A summary of the findings of the 19 simulation modelling studies included in the review that assessed the impact of taxes on diet is outlined in Table 1. The layout of Table 1 allows assessment of the impact of a tax on a given food on that food itself (own-PE) and other foods not actually taxed (cross-PE). The following types of taxes were assessed: (1) sweetened beverages (*n = *4 [8],[30],[43],[44]), (2) carbonated soft drinks (*n = *5 [32],[33],[37],[38],[58]), (3) saturated fat (*n = *5 [35],[36],[39],[40],[42]), (4) sugar (*n = *1 [45]), and (5) less healthy/junk foods (*n = *3 [36],[40],[41]).

There was substantial variability in outcomes assessed by the 19 studies (Table 1). However, three or more studies assessed the impact of a carbonated soft drink tax on the same food or nutrient outcome (*n = *4 for carbonated soft drink purchases and *n = *3 for energy [calories]). In addition, five studies assessed the impact of a saturated fat tax on saturated fat consumption (italicised in Table 1). Overall findings were pro-health, with the majority of studies in each category estimating lowered consumption of the taxed food.

**Figure 4:**
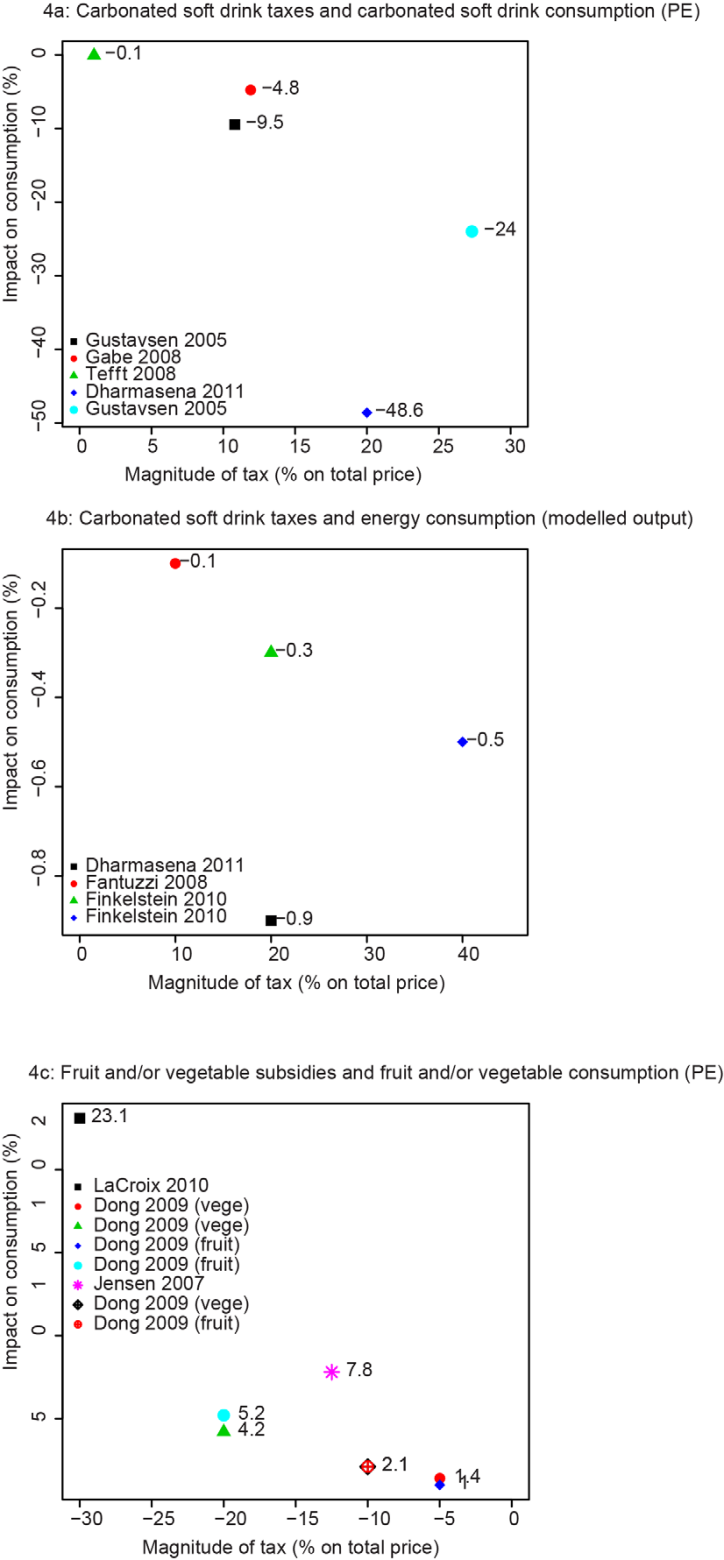
Relationships between fiscal pricing strategies and change in food consumption. (A) Carbonated soft drink taxes and carbonated soft drink consumption. (B) Carbonated soft drink taxes and energy consumption. (C) Fruit/vegetable subsidies and fruit/vegetable consumption.

The estimated mean own-PE, which represents the change in demand with a 1% change in price, for carbonated soft drinks was −0.93 (range, −0.06, −2.43) (Figure 4A). The corresponding modelled reduction in the amount of energy (calories) purchased resulting from a carbonated soft drink tax was −0.02% (range, −0.01%, −0.04%) for each 1% increase in price. Figure 4A illustrates that the relationship between carbonated soft drink taxes and reductions in consumption of carbonated soft drinks appears linear. Sensitivity analyses to explore the effect of studies contributing more than one PE value to the overall mean did not substantially change the own-PE for carbonated soft drinks (mean PE for sensitivity analysis, −0.94 [range, −0.06, −2.43]), but almost halved the modelled reduction in energy consumption (−0.014% [range, −0.01, −0.04%]). However, none of these studies were assessed as moderately high quality.

Only three of the five studies assessing the impact of a saturated fat tax on saturated fat consumption could be combined. This was due to differences in the format of taxes: three studies assessed flat rate taxes on major dietary contributors to saturated fat [35],[36],[40], and the remaining two studies assessed nutrient taxes per kilogram of saturated fat [39],[42]. For the three studies assessing a flat rate tax, the mean modelled reduction in saturated fat consumption in response to a 1% increase in price was −0.02% (range, −0.01%, −0.04%) energy from saturated fat (findings for one study [40] were converted from percentage reduction in saturated fat to percentage reduction in total energy from saturated fat assuming 12.8% energy consumed from saturated fat in the British population [59]). No studies contributed more than one value to the overall mean impact. In comparison, the two studies assessing nutrient taxes estimated slightly greater reductions of −1.11% and −1.35% of energy from saturated fat resulting from taxes of 14 and 7.9 Danish Krone per kilogram of saturated fat, respectively (€1.88/US$2.33 and €1.06/US$1.31, respectively). Four studies in this section were considered moderately high quality [36],[39],[40],[42]; removal of the lower quality study from the former pooled figure increased the mean reduction in saturated fat to −0.03% (range, −0.02%, −0.04%) energy from saturated fat. There was some evidence from three of the moderately high quality studies of unintended compensatory purchasing that could mitigate the potential health impacts of food taxes (because of a combination of change in expenditure budget and cross-PEs; Table 1). Specifically, saturated fat taxes were estimated to increase consumption of sodium (*n = *2 studies [36],[40]), energy (*n = *1 study [36]), and sugar (*n = *1 study [42]), and a tax on less healthy foods was estimated to increase consumption of saturated fat [36].

There were too few studies to estimate the pooled impact of any other tax scenarios on consumption of foods or nutrients.

#### Subsidies

Seven studies estimated the impact of five subsidies, as outlined in Table 2: (1) all soft drinks (*n = *2 [33],[46]), (2) fruits and vegetables (*n = *2 [39],[47]), (3) fruits and vegetables and fish (*n = *1 [45]), (4) fibre (*n = *1 [39]), and (5) all healthier products (*n = *1 [51]).

**Table 2:** Summary of simulation modelling studies assessing the effects of subsidies on food/nutrient consumption (*n = *7 studies).

Subsidised Food or Nutrient	Measured Food or Nutrient	Impact of Subsidy			Mean (Standard Deviation) PE^a^
		Number of Studies Reporting Increased Consumption	Number of Studies Reporting No Impact	Number of Studies Reporting Decreased Consumption	
**Soft drinks**	All soft drinks	2 [33],[46]			
**Fruits and vegetables**	*Fruits and vegetables*	*3 * [39],[47],[51]			*−0.35 (0.22)*
	Flour	**1 [39]**			
	Fibre	**2 [42],[39]**			
	Milk			**1 [39]**	
	Butter and fat			**1 [39]**	
	Cheese			**1 [39]**	
	Eggs			**1 [39]**	
	Fish			**1 [39]**	
	Total fat			**1 [39]**	
	Saturated fat			**2 [39],[42]**	
	Meat		**1 [39]**		
	Sugar			**1 [42]**	
**Fibre**	Fruits and vegetables	**1 [39]**			
	Flour	**1 [39]**			
	Fibre	**2 [39],[42]**			
	Milk			**1 [39]**	
	Butter and fat			**1 [39]**	
	Cheese			**1 [39]**	
	Eggs			**1 [39]**	
	Fish			**1 [39]**	
	Total fat			**1 [39]**	
	Saturated fat			**2 [39],[42]**	
	Sugar	**1 [42]**			
**Fruits and vegetables, fish**	Bread		1 [45]		
	Meat	1 [45]			
	Fish	1 [45]			
	Fruits and vegetables	1 [45]			
	Sugar and sweets		1 [45]		
**All healthier products**	All healthier products	1 [51]			
	Fruits and vegetables	1 [51]			
	Neutral products			1 [51]	
	Less healthy products			1 [51]	

Bold indicates studies that were considered moderately high quality (see Text S2). Italicised rows represent scenarios where sufficient studies were available to quantitatively aggregate (≥3 similar studies).

a Mean PE value calculated only where ≥3 studies per major category.

There was substantial variability in outcomes assessed by the seven simulation studies in this category (Table 2). However, three studies assessed the impact of eight fruit and vegetable subsidies of varying magnitude on consumption of fruits and vegetables (italicised in Table 2). One study was considered moderately high quality [39]; the finding of this study was consistent with that of the lower quality studies, i.e., overall findings were pro-health and the estimated mean own-PE was −0.35 (range, −0.21, −0.77). The standard way of presenting PEs is to present the percentage reduction in consumption that would be expected with each 1% price *increase*. However, in modelling a subsidy on fruits and vegetables, price will *decrease*, and thus purchases will likely increase, i.e., in this case a 0.35% increase in fruit and vegetable purchases for each 1% reduction in price. Figure 4B illustrates that the relationship between the magnitude of the fruit and vegetable subsidy and the increase in consumption of fruits and vegetables appears linear. Sensitivity analyses to explore the effect of studies contributing more than one PE value to the overall mean increased the own-PE (mean sensitivity PE, −0.54 [range, −0.23, −0.77]).

There were too few studies to determine the pooled impact of any other food subsidies on foods or nutrients. However, two moderately high quality studies provide some evidence that a subsidy on foods high in fibre decreases consumption of saturated fat [39],[42] (Table 2). There was also some suggestion of unintended compensatory purchasing, with one moderately high quality study indicating that a fruit and vegetable subsidy may result in decreased fish consumption, and a fibre subsidy in decreased fish and increased sugar consumption [39].

#### Tax and subsidy combinations

The eight simulation modelling studies that assessed the impact of combinations of food taxes and subsidies on food and nutrient consumption evaluated ten different combinations of taxes and subsidies, as outlined in Table 3.

**Table 3:** Summary of simulation modelling studies assessing the impact of tax and subsidy combinations on food and nutrient consumption (*n = *8 studies).

Taxed and Subsidised Foods and Nutrients	Measured Foods and Nutrients	Impact of Combined Tax and Subsidy		
		Number of Studies Reporting Increased Consumption	Number of Studies Reporting No Impact	Number of Studies Reporting Decreased Consumption
**Saturated fat tax, sugar tax, fibre subsidy**	Milk			**1 [39]**
	Butter and fat			**1 [39]**
	Cheese			**1 [39]**
	Meat			**1 [39]**
	Fish			**1 [39]**
	Sugar			**1 [39]**
	Total fat			**1 [39]**
	Saturated fat			**1 [39]**
	Eggs	**1 [39]**		
	Flour	**1 [39]**		
	Fruits and vegetables	**1 [39]**		
	Fibre	**1 [39]**		
**Total fat tax, sugar tax, fruit and vegetable subsidy**	Milk			**1 [39]**
	Butter and fat			**1 [39]**
	Cheese			**1 [39]**
	Eggs			**1 [39]**
	Meat			**1 [39]**
	Fish			**1 [39]**
	Sugar			**1 [39]**
	Total fat			**1 [39]**
	Saturated fat			**1 [39]**
	Flour	**1 [39]**		
	Fruits and vegetables	**1 [39]**		
	Fibre	**1 [39]**		
**Less healthy food tax, fruit and vegetable subsidy**	Energy			**1 [40]**
	Saturated fat			**1 [40]**
	Salt			**1 [40]**
	Fruits and vegetables			**1 [40]**
**Bakery product tax, ready meals tax, healthy breads and cereals subsidy**	Bread and cereals	1 [61]		
	Bakery products			1 [61]
	Ready meals			1 [61]
	Fibre	1 [61]		
	Energy	1 [61]		
	Salt	1 [61]		
	Sugar	1 [61]		
	Total fat	1 [61]		
	Added sugar	1 [61]		
	Saturated fat			1 [61]
**Grain tax, fibre subsidy**	Bread and cereals	1 [61]		
	Bakery products			1 [61]
	Ready meals			1 [61]
	Fibre	1 [61]		
	Energy	1 [61]		
	Salt	1 [61]		
	Total fat	1 [61]		
	Sugar			1 [61]
	Saturated fat			1 [61]
**Saturated fat tax, fibre subsidy**	Bread and cereals	1 [61]		
	Bakery products			1 [61]
	Ready meals			1 [61]
	Fibre	1 [61]		
	Energy	1 [48]		
	Salt	1 [61]		
	Total fat	1 [61]		
	Sugar	1 [61]		
**Total fat tax, fruit and vegetable subsidy**	Foods high in total fat			1 [49]
	Protein		1 [50]	
	Total fat			1 [50]
	Saturated fat			1 [50]
	Monounsaturated fat			1 [50]
	Polyunsaturated fat			1 [50]
	Sugar	1 [50]		
	Energy			1 [50]
	Cholesterol			1 [50]
	Sodium			1 [50]
	Fibre	1 [50]		
	Fruits and vegetables	1 [50]		
**Meat tax, butter tax, Cheese tax, fruit and vegetable subsidy, grain subsidy**	Saturated fat			**1 [42]**
	Sugar	**1 [42]**		
	Fibre	**1 [42]**		
**Saturated fat tax, sugar tax, fibre subsidy**	Saturated fat			**1 [42]**
	Sugar			**1 [42]**
	Fibre	**1 [42]**		
**Less healthy food tax, healthy food subsidy**	Fruits and vegetables	1 [51]		
	Healthy foods	1 [51]		
	Neutral foods			1 [51]
	Less healthy foods			1 [51]
**Sugar tax, fruit and vegetable subsidy, fish subsidy**	Bread			1 [45]
	Meat	1 [45]		
	Fish	1 [45]		
	Fruits and vegetables	1 [45]		
	Sugar and sweets			1 [45]

Bold indicates studies that were considered moderately high quality (see Text S2).

Most of the tax and subsidy combinations examined included a total fat and/or saturated fat tax and a fibre/grain and/or fruit and vegetable subsidy (Table 3). There was substantial variation in the outcomes assessed for each tax and subsidy combination, and thus it was not possible to pool the findings of studies to determine potential impacts on food and nutrient consumption. However, the two moderately high quality studies included in this section of the review support tax and subsidy combinations as being pro-health: Nnoaham and colleagues estimated that a less healthy food tax combined with a fruit and vegetable subsidy would decrease purchases of energy, saturated fat, and sodium [40], and Smed and colleagues reported that a saturated fat and sugar tax combined with a fibre subsidy decreased purchases of saturated fat and sugar while increasing fibre purchases [42] (Table 3). However, Smed et al. [42] estimated that some unintended compensatory buying would occur, with the less healthy food tax and fruit and vegetable subsidy combination resulting in an overall decrease in fruit and vegetable purchases.

### Objective 2: Impact on Health and NCDs

#### Taxes

The 16 simulation modelling studies that assessed the impact of food taxes on health and disease evaluated ten types of taxes, as outlined in Table 4.

**Table 4:** Summary of simulation modelling studies assessing the impact of taxes on health/disease (*n = *16 studies).

Taxed Food or Nutrient	Measured Risk Factor or Disease	Impact of Tax		
		Number of Studies Reporting Improved Health/Burden of Disease	Number of Studies Reporting No Impact	Number of Studies Reporting Worsened Health/Burden of Disease
**Dairy/saturated fat**	Body weight		1 [31]	
	Serum cholesterol	**1 [36]**		1 [35]
	IHD			1 [35]
	Number of deaths avoided			1 [35]
	Mortality from IHD	**1 [36]**		
	Mortality from stroke	**2 [36],[40]**		
	Annual deaths from CVD	**1 [36]**		**1 [40]**
	Annual deaths from CHD			**1 [40]**
	Annual deaths from cancer	**1 [40]**		
**All foods**	Number of lives saved			**1 [52]**
**Less healthy and intermediate healthy foods**	Number of lives saved			**1 [52]**
**Less healthy foods**	Number of lives saved			**1 [52]**
**Sugar-sweetened beverages**	Body weight			1 [37]
**Soft drinks**	Body weight			1 [13],[56]
	Overweight			2 [43],[53]
	Obesity		1 [53]	1 [43]
	BMI		**1 [55]**	1 [53]
	Weight loss			1 [43]
**Bacon, ice cream, and white sugar**	BMI			1 [54]
**Snack foods (potato chips and salty snacks)**	Weight loss			1 [34]
**Less healthy/junk food**	Serum cholesterol	**1 [36]**		
	Mortality from IHD	**1 [36]**		
	Mortality from stroke	**1 [40]**		**1 [36]**
	Annual deaths from CVD			**2 [36],[40]**
	Annual deaths from CHD			**1 [40]**
	Annual deaths from cancer	**1 [40]**		
	Body weight			1 [41]
**Food away from home**	Body weight	1 [56]		
**Sugar**	Body weight	1 [45]		
	Incidence of type 2 diabetes	1 [45]		
	Incidence of CHD	1 [45]		

Bold indicates studies that were considered moderately high quality (see Text S2). BMI, body mass index; IHD, ischemic heart disease.

There was substantial variation in the outcomes assessed for each type of tax, and thus it was not possible to pool the findings of studies to determine potential impacts on health and disease. Despite the majority of studies in this section of the review suggesting that impacts of food/nutrient taxes would be pro-health, there were also a number of modelling studies that estimated that taxes may affect health outcomes adversely, particularly taxes focusing on dairy/saturated fat and junk food (Table 4). The three moderately high quality studies in this section estimated that (1) a dairy/saturated fat tax may increase mortality from CVD and coronary heart disease (CHD) (*n = *1 study [40]), and (2) a less healthy/junk food tax may increase overall mortality (*n = *1 study [60]) and mortality from stroke and CVD (*n = *2 studies [36],[40]). The potential adverse health consequences estimated as a response to these taxes is due to compensatory purchasing via cross-PEs. For example, in the case of a tax on dairy/saturated fat, the increase in price may reduce purchases of dairy/saturated fat through the own-PE. However, compensatory purchasing may occur that undermines the intention of the tax. In this case, purchases of food substitutes for dairy/saturated fat may increase (via cross-PEs). If these foods are just as unhealthy as the taxed food, or even more so, the result is that the diet is not improved, or in some circumstances may even be worse. For example, bread may be seen as suitable substitute for yoghurt (dairy) as a snack, but if the bread chosen is high in salt, then risk of CVD may inadvertently increase.

#### Subsidies

Three simulation modelling studies estimated the impact of food subsidies on health and disease [45],[56],[57]. These studies assessed subsidies on (1) fruits and vegetables (*n = *2 [57]), (2) fruits and vegetables and fish (*n = *1 [45]), and (3) diet soft drinks (*n = *1 [56]).

Although the majority of these modelling studies estimated that food subsidies would improve health and reduce NCDs, there were too few studies (and no moderately high quality studies) to pool the findings for any subsidy regimens or disease outcomes (Table 5).

**Table 5:** Summary of simulation modelling studies assessing the impact of subsidies on health/disease (*n* = 3 studies).

Subsidised Food or Nutrient	Measured Risk Factor or Disease	Impact of Subsidy		
		Number of Studies Reporting Improved Health/Burden of Disease	Number of Studies Reporting No Impact	Number of Studies Reporting Worsened Health/Burden of Disease
**Fruits and vegetables**	Number of cases of CHD prevented	1 [57]		
	Number of cases of ischemic stroke prevented	1 [57]		
	Body weight			1 [56]
**Fruits and vegetables, fish**	Body weight			1 [45]
	Incidence of CHD			1 [45]
	Risk of CHD	1 [45]		
	Cardiovascular mortality	1 [45]		
**Diet soft drinks**	Body weight	1 [56]		

#### Tax and subsidy combinations

The four modelling studies assessing the impact of food tax and subsidy combinations on health and disease evaluated three combinations of taxes and subsidies, as summarised in Table 6: (1) a less healthy food tax combined with a fruit and vegetable subsidy (*n = *2 [40],[52]), (2) a saturated fat tax combine with a fruit and vegetable subsidy (*n = *1 [50]), and (3) a sugar tax combined with a fruit and vegetable and fish subsidy (*n = *1 [45]).

**Table 6:** Summary of simulation modelling studies assessing the impact of tax and subsidy combinations on health/disease (*n = *3 studies).

Taxed and Subsidised Foods and Nutrients	Measured Risk Factor or Disease	Impact of Combined Tax and Subsidy		
		Number of Studies Reporting Improved Health/Burden of Disease	Number of Studies Reporting No Impact	Number of Studies Reporting Worsened Health/Burden of Disease
**Less healthy food tax, fruit and vegetable subsidy**	Number of lives saved	**1 [52]**		
	Annual deaths from CHD	**1 [40]**		
	Annual deaths from stroke	**1 [40]**		
	Annual deaths from cancer	**1 [40]**		
	Annual deaths from all CVD	**1 [40]**		
**Saturated fat tax, fruit and vegetable subsidy**	Gastric cancer	1 [50]		
	Lung cancer	1 [50]		
	CVD	1 [50]		
	CHD	1 [50]		
	Ischemic stroke	1 [50]		
	All chronic disease	1 [50]		
**Sugar tax, fruit and vegetable subsidy, fish subsidy**	Body weight	1 [45]		
	Incidence of type 2 diabetes	1 [45]		

Bold indicates studies that were considered moderately high quality (see Text S2).

Although all of the modelling studies in this category of the review estimated effects to be pro-health (regardless of the outcome measured), the evidence was too limited to evaluate the pooled impact of any tax and subsidy combination on any health or disease outcome (Table 6). Nonetheless, two of the three studies in this section were moderately high quality and estimated positive impacts of a tax on less healthy foods combined with a fruit and vegetable subsidy (i.e., fewer overall premature deaths, and fewer premature deaths from CVD, CHD, stroke, and cancer) [40],[52]. No moderately high quality studies provided any evidence of unintended impacts on health.

### Objective 3: Differences in Impact by Socio-Economic Group

Of the 32 simulation modelling studies included in the review, nine specifically reported differential PEs by socio-economic group [29],[31],[42],[44],[47],[48],[51],[53],[57], and a further five [13],[38], undertook regression or sensitivity analyses to estimate impact of food taxes and subsidies separately by socio-economic position. The findings of these 14 studies are summarised in Table 7.

**Table 7:** Summary of simulation modelling studies assessing the impact of taxes on food and nutrient consumption, health, and disease of lower socio-economic population groups (*n = *14 studies).

First Author (Year) [Reference]	Impact for Lower Socio-Economic Group^a^			Relative Impact Compared with Higher Socio-Economic Groups			Key Points
	Pro-Health	No Impact	Counter to Health	Greater Impact for Lower Socio-Economic Group	No Difference	Smaller Impact for Lower Socio-Economic Group	
**Allais (2010) [29]**	✓			✓			Used differential PEs by socio-economic group. Positive impact on food purchases for modest and well-off, although greater impacts for modest.
Cash (2005) [57]	✓					✓	Used differential PEs by socio-economic group. Fewer lives saved for lower income compared with middle- and high-income groups.
Chouinard (2007) [31]	✓				✓		Used differential PEs by socio-economic group. Small positive impact on food purchases, but no impact on health. Similar findings across income groups, therefore only overall reported.
Dong (2009) [47]	✓			NR	NR	NR	Used differential PEs by socio-economic group. Positive impact on food purchases.
Fantuzzi (2008) [13]	✓			✓			Positive impact on food purchases and health, with greater PEs for lower income groups.
Finkelstein (2010) [38]		✓				✓	Varied impact on food purchases. Any positive impacts generally driven by middle-income group.
Fletcher (2008) [53]	✓				✓		Used differential PEs by socio-economic group. Positive impacts on health greater for black and white individuals than for Hispanic individuals.
LaCroix (2010) [51]		✓			✓		Used differential PEs by socio-economic group. Varied impact on food purchases for low-income group. Similar impact for low- and high-income groups.
**Nnoaham (2009) [40]**	✓				✓		Positive impact on food purchases and health similar across income groups.
Nordstrom (2010) [48]	✓				✓		Used differential PEs by socio-economic group. Positive impact on food purchases similar in magnitude across income groups.
Sassi (2009) [49]	✓			✓			Greater positive impact on food purchases for lower socio-economic group.
**Smed (2007) [42]**	Varied			Varied			Used differential PEs by socio-economic group. Varied impact on food purchases, with some impacts greater for lower income groups.
Tefft (2008) [58]	✓				✓		Positive impact on food purchases similar in magnitude across socio-economic groups.
Zhen (2011) [44]	✓			✓			Used differential PEs by socio-economic group. Positive impact on food purchases greater for low-income groups.

No trials included in this analysis assessed food subsidies. Bold indicates studies that were considered moderately high quality (see Text S2).

NR, not reported.

Overall, pricing policies appeared to result in improved food and nutrient consumption and health benefits for lower socio-economic groups. This finding was supported by 11/14 studies, including one moderately high quality study [40]. However, many noted that such taxes would be regressive, with the financial burden falling predominantly on the lowest income groups, in which individuals spend the largest proportion of their total budget on food [29],[31],[44]. Nonetheless, 4/14 studies estimated that pricing policies would result in greater health benefits for lower socio-economic compared with higher socio-economic groups, and thus have the potential to reduce health inequalities [13],[29],[44],[49]. Half of the studies in this section estimated that relative impacts would be similar across socio-economic groups (including two moderately high quality studies), which, given that lower socio-economic groups usually have higher NCD burden, translates into a greater absolute impact in lower socio-economic groups. For example, if the relative reduction in CVD is the same for higher and lower income groups (e.g., 10% in each group), but the prevalence of CVD in the lower income group is higher (e.g., 40% compared with 20% for those with higher incomes), the lower income group will stand to receive a greater absolute reduction in CVD (0.1×40% = 4% for the lower income group versus 0.1×20% = 2% for the higher income group). The remaining three studies estimated poorer health outcomes for lower socio-economic groups [38],[42],[57] (one study did not report differences in magnitude by socio-economic group [47]). Findings for the two moderately high quality studies were mixed, with one estimating improved dietary and health outcomes (but no relative differences in impact across socio-economic groups) [40], and the other estimating varied impacts on dietary and health outcomes (with some relative differences by socio-economic group) [42].

## Discussion

Notwithstanding the low to moderate quality of the majority (27/32) of the included studies, the overall finding of this review is that pricing strategies have the potential to produce changes in population food consumption. A summary of key findings is provided in Box 1.

This review carefully examined the quality of simulation models, and only 22% (7/32) of included studies were considered moderately high quality. Less than half (13/32) of included studies used a complete food demand system encompassing both own- and cross-PEs. The lack of inclusion of cross-PEs in particular may have affected the results of this review. For example, if less healthy foods are relatively price-inelastic (i.e., increases in price do not greatly influence amounts purchased of the targeted food items), then the remaining budget available to spend on other foods is smaller, and, because of the balance of cross-PEs, purchasing shifts away from healthier foods such as fruits and vegetables to poorer quality foods high in saturated fat and sodium. If a larger number of studies had included cross-PEs, it may have been possible to quantitatively assess these unintended effects. Indeed, we would recommend that simulation studies that include only own-PEs be treated with caution.

Nonetheless, a particular aim of this review was to collate and quantitatively summarise the best evidence from simulation modelling studies regarding the association between food pricing strategies, food consumption, health, and NCDs. Therefore, although the pooled estimates are based on lower quality studies, the estimates can be improved upon as more relevant, higher quality research becomes available. Plotting the estimated impact on consumption resulting from food taxes and subsidies of various magnitudes (Figure 4) produces a linear trend, suggesting that, to a point, overall, the larger the magnitude of the tax or subsidy, the larger the impact on consumption (in the desired direction). Figure 4's scatter plots effectively illustrate the mean PE. As more evidence becomes available, more points could be added to these scatter plots to help to determine the potential shape of the PE curve.

An updated literature search to October 2012 identified three additional studies meeting the inclusion criteria for this review [62]–[64]. All three focussed on sugar-sweetened beverages, with two exploring the impact of taxes [63],[64], and one the impact of a tax reform [62]. Consistent with previous research, these studies failed to assess impacts on the complete food demand system, although all took into account compensatory purchasing of other types of beverages via cross-PEs. Because of the variability in the metrics used by these studies to tax soft drinks and assess the outcomes, it was not possible to add these studies to the scatter plots illustrating the PE curve. However, the results of these newer studies align with those of similar studies included in the review [8],[45]: a penny-per-ounce sugar-sweetened beverage tax in the United States was estimated to reduce purchases of these beverages by 15% in adults and prevent 26,000 premature deaths between 2010 and 2020 [64], a 39% sugar-sweetened beverage tax in the United States was estimated to decrease sugar purchases by 10% [63], and a reform of the European Union sugar policy in France to effectively decrease the price of regular soft drinks by 3% was estimated to raise purchases of these drinks by 1 litre per person per year [62].

The majority of studies in this review failed to take into account that own- and cross-PEs are difficult to calculate accurately and precisely and thus that error/variation must be taken into account in the modelling process (error and appropriate reflection of uncertainty is important in modelling). Reporting of statistical variance around a point estimate, and discussion of any suspected systematic errors would provide some idea of the uncertainty of the own- and cross-PEs (and hence uncertainty of the overall findings of the simulation model). Furthermore, no studies included in the review attempted to validate the epidemiological model used to estimate impacts on consumption, health, and disease. Validation is important because underlying model structure and assumptions vary widely between models and are associated with uncertainty; without validation or comparison of findings with other models, it is difficult to determine whether findings are real, or in fact artefacts of the model itself.

Most studies in this review (25/32; 78%) failed to estimate the uncertainty of model findings. Uncertainty arises from the model structure and variation in the model inputs, including food consumption data, food prices, relative risks, and PEs. Uncertainty arising from the model structure is more difficult to estimate, but uncertainty arising from inputs may be dealt with using Monte Carlo simulations [65]. In summary, the quality of future simulation modelling studies could be vastly improved by (1) using a complete demand system including own- and cross-PEs, (2) estimating the uncertainty around model outputs, and (3) attempting to validate epidemiological models. The impact of ten key quality indicators for simulation models is further discussed in Text S2.

Another limitation of this review is that it is possible that publication bias is present. Such bias is common in systematic reviews and is often assessed using a funnel plot [66]. However, the studies in this review were not randomised controlled trials, the pricing strategies and outcomes were highly variable, and most studies failed to report variability around the point estimate. Therefore, it was not possible to create a funnel plot. Bias may also have been introduced via the search strategy, which was limited to articles and reports published in English and conducted in member countries of the OECD [67]. Nonetheless, the titles of all non-English-language studies were available in English and were thus reviewed; none were likely to meet the inclusion criteria for this review. Further, a number of the included studies were from countries where English is not the primary language, i.e., France (*n = *2), Norway (*n = *1), Denmark (*n = *2), Finland (*n = *1), and Sweden (*n = *1), and thus any impact of this potential bias on study findings is likely to have been minimal. With respect to the exclusion of non-OECD countries, only two studies, both from Egypt, were excluded. Furthermore, one of these studies did not assess any of the outcomes pre-specified for this review [28], and the other assessed a food subsidy programme that aimed to reduce under- rather than over-nutrition [27]. Nonetheless, it is notable that included studies were from middle- to high-income countries, and thus the findings of this review may not be generalisable to low-income countries.

The findings of this review support those of a previous systematic review undertaken by Thow and colleagues (*n = *16 simulation modelling studies to 2009) [20]. However, the findings of the current review indicate smaller changes in diet, health, and disease resulting from food taxes and subsidies compared with those suggested by Thow et al. This is likely due to many studies in the earlier review failing to take into account shifts in consumption of non-targeted foods (using cross-PEs). Inclusion of cross-PEs enables assessment of unintended compensatory or displacement purchasing that can undermine the health objective of the tax or subsidy. Larger cross-PEs indicate that non-targeted foods are suitable substitutes for targeted/taxed/subsidised foods, and thus larger cross-PEs mean a higher likelihood of compensatory purchasing. If such purchasing involves a less healthy dietary replacement, then it is likely to produce effects that are counter to health. For example, in the moderately high quality study by Mytton et al. [36], authors assessed the impact of a 17.5% tax on principal sources of saturated fat in the British diet. The tax was estimated to reduce percentage energy consumed from saturated fat (by −0.13%), but through unintended compensatory purchasing, salt intake was also estimated to increase by 5.3%. However, if compensatory purchasing involves a healthier replacement, then large positive impacts on health are likely to be observed. The impacts of compensatory purchasing are of particular importance for lower income households, where both own- and cross-PEs are commonly larger than for households with higher income [13],[68].

Fourteen studies in this review evaluated the potential impacts of pricing strategies for lower socio-economic population sub-groups, which are most at risk of having poor dietary intakes and NCDs. The majority of these studies (11/14) estimated that fiscal policies would result in absolute improvements in dietary outcomes for lower socio-economic groups, although only four studies estimated relatively greater health benefits for lower compared with higher socio-economic groups, and thus improved health equity. Findings for the two moderately high quality studies were mixed, and several authors noted that taxes would be regressive. Whether taxes, subsidies, or combinations of both result in greater dietary and health improvements in lower compared with higher socio-economic groups is important to consider. If PEs are greater among low-income population sub-groups, then negative financial implications will be at least partially offset by greater improvements to health and an overall effect of decreasing health inequities (as has been observed for tobacco taxes [69]). Furthermore, overall health benefits for lower socio-economic groups may be greater if food taxes are combined with other fiscal policies to counterbalance increased food costs (such as lower income tax for low-income earners and increases in welfare benefits).

Although food pricing strategies show promise for improving population health, this review highlights several important areas for future research. First, the association of pricing strategies with non-targeted food consumption must be evaluated through inclusion of cross-PEs that are appropriate and valid for the population being investigated. This would be particularly valuable with respect to soft drink and saturated fat taxes, and fruit and vegetable subsidies. Second, effects of food pricing strategies must be assessed for lower socio-economic population groups, which are most at risk of NCDs, particularly in countries where there are large health inequalities. Third, long-term health and NCD mortality must be assessed. The cost-effectiveness of food pricing strategies is also particularly important to investigate if such strategies are to be considered feasible for implementation. Moreover, there are many pragmatic issues that need to be addressed by future research. These include whether it is best to apply taxes/subsidies at the point of sale or point of production (i.e., sales or excise taxes), which magnitude and combination of taxes and/or subsidies will be most effective, whether price changes should be applied at a flat rate (e.g., increasing the price of butter at the point of sale) or at a rate per nutrient/volume of food (e.g., increasing the price of butter by an amount per gram of saturated fat) [12],[20], and the percentage of tax or subsidy that reaches the consumer (tax pass through). Given the limitations of the current evidence, robust evaluations must be planned when food pricing policies are implemented by governments.

It must be noted that the impact of any given food tax or subsidy is likely to differ by country (i.e., heterogeneity of impact by context), depending on factors such as the type of tax system implemented, health status, co-existent marketing, cultural norms, expendable income, and the social role of food [70]. Unfortunately, the use of different types of datasets and epidemiological models by authors of papers in this review meant that it was not possible to compare the potential effects of specific food pricing strategies by population or country of interest. Furthermore, evidence for low-income countries is largely absent. While we currently have far too few studies to assess how much impacts vary by context, we must expect that they will and evaluate future research in that light.

The best evidence for the effectiveness of food pricing strategies may be the results of “natural experiments”. Differential application of goods and services tax or value added tax currently occurs in several countries, including Australia, Canada, France, and the United Kingdom. However, the primary purpose of these differential taxes is not to promote health. Nonetheless, four such natural experiments were evaluated in the current review, one of which was undertaken in Ireland [46], and three in the United States [8],[32],[55]; all focused on soft drinks or sugar-sweetened beverages. Three of these studies assessed effects on food or nutrient consumption, and all three studies reported impacts in the desired direction [8],[32],[46] (two assessed effects on health: one found no impact [55] and the other found a positive impact [8]). Several countries currently have differential health-related food taxes [71]. Most recently (2011 or 2012), Denmark implemented a €2.41 levy per kilogram of saturated fat (for products ≥2.3% saturated fat; the tax increases with amount of saturated fat), France applied a €0.036 per litre tax on sweetened beverages [72], and Hungary introduced a 10-forint tax (€0.04) per item on foods high in total fat, sugar, and salt [73]. However, no evaluation of the impact of these taxes on food purchases and health has yet been published.

In conclusion, this review systematically summarises the best evidence available to date on the association between food pricing strategies, food consumption, health, and NCDs. We make recommendations regarding better design, assessment of quality, and reporting of future simulation studies. Furthermore, we present pooled estimates of effect. The current evidence suggests food pricing strategies show potential for changing population diets and long-term health and disease outcomes; quantitative analyses indicate own-PEs of demand for carbonated soft drinks and fruits and vegetables, which are reasonably elastic on a population scale (−0.93 [range, −0.06, −2.43] and −0.35 [range, −0.21, −0.77], respectively), and a modelled −0.02% reduction (range, −0.01%, −0.04%) in energy from saturated fat for each 1% price increase. Based on these PE estimates, a 10% increase in the price of soft drinks could decrease consumption by −0.6% to −24.3%; conversely, a 10% decrease in the price of fruits and vegetables could increase consumption by between 2.1% to 7.7%. Nonetheless, high-quality evidence is lacking, particularly with regard to the unintended effects of compensatory purchasing and the potential impacts on health equity, long-term health, and NCD mortality. Moreover, cost-effectiveness and pragmatic issues associated with the implementation of food pricing strategies must also be addressed. Robust evaluations built into the implementation of food pricing policies would help to answer some of these questions and engender confidence that such strategies will provide positive effects on population diets and reduce the global burden of NCDs.

## Supporting Information

Text S1 **PRISMA checklist.**
(DOC)

Text S2 **Quality and characteristics of included studies.**
(DOCX)

## Abbreviations

CHD: coronary heart disease
CVD: cardiovascular disease
NCD: non-communicable disease
OECD: Organisation for Economic Co-operation and Development
PE: price elasticity
RCT: randomised controlled trial
