# Supplementary material for: Food Pricing Strategies, Population Diets, and Non-Communicable Disease: A Systematic Review of Simulation Studies
Source: PLoS Med. 2012 Dec 11;9(12):e1001353. doi: 10.1371/journal.pmed.1001353 (PMC3519906; doi:10.1371/journal.pmed.1001353)
Supplement: Text S2 — Quality and characteristics of included studies. (DOCX) [file pmed.1001353.s002.docx]

**Quality of simulation studies**

This appendix presents a quality assessment of the included studies. Each column of the table below pertains to one characteristic. We consider major columns in turn:

1. *Complete food demand system.* Econometric models to estimate price elasticties can include one or more food categories. However, a complete demand system includes more than one food category and encompasses all or most (at least 70%) types of foods consumed. For example, a system that includes only milk, cheese, eggs, and yoghurt is not a complete demand system. However, a system that includes all food groups except restaurant and take-away foods may be considered a complete demand system. A complete system is preferable for modelling health impacts, as it means that the impact of an intervention on all food groups can be estimated (via cross- price elasticities; PEs). A complete demand system is not usually easy to estimate because of data limitations (e.g. food groupings of the pricing data not matching with those of the consumption data). Moreover, a complete demand system that includes very few food categories (for example, food consumed at home and restaurant and take away foods) is not necessarily better than a partial system including several food categories; we consider at least eight food groups as a minimum. Nonetheless, own- and cross-PEs generated from a complete system are generally lower in magnitude compared to those generated from a partial demand system, and using PEs estimated from a partial demand system may produce misleading findings (as a result of incomplete information of the impact of the intervention on all food groups and the high magnitude of PEs).
2. *PE input data short- or long-run*. We assume that any tax or subsidy will be a long-run policy instrument, i.e. it will be implemented for years, not ‘turned on and off’. Econometric estimation methods can be ‘turned’ to either estimate the short- or long-run price elasticity. Features of econometric studies that cause the estimation of PEs to be more ‘long-run’ include: using long-run data (i.e., more than two years); using data which has been collected continuously or very frequently (i.e. at least monthly) in the same population (for example, panel data), or data which has been collected in large samples of different populations over a number of years (i.e. at least five years; for example, aggregation of five years of annual cross-sectional national survey data). Time series data are not usually considered long-run because although collected in the same population over time, there are usually few data collection points. Long-run PEs are also associated with stock and habit formation variables in the demand equations. [[1](#_ENREF_1)]. Classifying criteria for long- or short- run PEs in this review were first based on the definition of long/short-run PEs by the author(s). If the PEs reported were not defined by the author(s), stock and habit formation variables in the demand equations were searched for. If none were found, the PEs were classified according to data period as follows: a) data period was longer than five years: long-run PEs; b) data period was longer than two years and data were collected monthly: long-run PEs; c) studies that used average PEs from the literature: long-run PEs; and d) all the remaining cases of PEs were classified as short-run PEs.
3. *Own and cross-PEs.* For a full estimation of health impacts, cross-PEs are required (preferably with stated random error or uncertainty). Cross-PEs are useful in determining the impact of a pricing intervention on both targeted and non-targeted food groups. Cross-PEs are generally small in magnitude compared to that of own-PEs. The estimations of cross-PEs could be impossible if there is little variation in the relative price of many foods [[2](#_ENREF_2)].
4. *Differential PEs by socio-economic group.* Another relevant public health consideration is whether PEs vary by social grouping. For example, it is now well accepted that younger and poorer smokers are more responsive to tobacco price increases, i.e., the PE varies by the person’s socio-economic position[[3](#_ENREF_3)]. Empirically determining whether PEs vary by social grouping is challenging. However, it seems a reasonable starting position to assume that PEs will often be greater for those on lower incomes. PEs by socio-economic group are useful for modelling equity impacts on health.
5. *Source of PEs valid and appropriate.* Pricing intervention modelling can be very sensitive to PEs. Meanwhile, PEs may vary considerably depending on data quality, econometric models, and demand equations. Sometimes the PEs for particular foods of interest for a given country might not be available. Here we considered whether the source of PEs was valid and appropriate based on the following criteria: relevant country (consistent with consumption data), standard error reported, low random error of estimates (whether confidence intervals were small i.e. considerably lower than mean values), sufficient variation in price (based on data period and frequency, e.g. data period longer than five years, and whether data were reported annually, quarterly, or monthly).
6. *Uncertainty of PEs estimate included in overall model uncertainty analyses.* There are many sources of uncertainty in PE estimates yet they appear to be afforded insufficient attention in their application to epidemiological models and public health policy advice. These include: the nature and quality of the underlying data, food grouping problems, the econometric model and demand functions. Because modelling results could be very sensitive to PEs, the specification of uncertainty about the input PEs that feed through into output uncertainty (e.g. the amount that diet changes) in overall model uncertainty analyses will help to inform decision makers better. In our assessment, a model was considered to include price elasticity uncertainties if PEs used in the simulation were randomly generated from a distribution or a Monte Carlo simulation.
7. *Source of consumption, prevalence, and mortality data appropriate:* Due to population differences in income, food culture, health, and disease, the impacts of pricing interventions are likely to differ by country. While valid and appropriate food price elasticity values will hopefully capture the majority of differences in consumption, it is important that input data in the model are valid and relevant for the country of interest. Here we considered whether: the country consumption, prevalence, and mortality data were sourced from was consistent that of the price elasticity data, whether the number of participants included in these data sets was sufficient (n≥1,000), and whether the survey sample was considered representative of the country of interest.
8. *Consumption data collected/projected over time :* Foods and nutrients consumed by individuals vary over time, and thus more than one day/time point of data collection generally provides more robust data regarding food consumption (depending on the size of the sample). We considered time series (collected at several time points in the same population); panel and aggregate national level sales or consumption data (collected continuously) to be collected/projected over time. Cross sectional data (collected at one point in time from a unique population) were not considered to be collected/projected over time unless two or more such data sets were combined.
9. *Model validated:* Simulation models investigating the impact of food pricing interventions on changes in food consumption vary widely in terms of their components and the underlying algorithms or risk ratios used to estimate resulting health impacts. Validation of a simulation model is difficult and not often reported. However, some idea of the validity of a model can be achieved by comparing the results of modelling across one or more different model structures (using the same intervention, PEs and consumption data).
10. *Uncertainty of model addressed:* There are many sources of uncertainty in simulation models, including: in estimation of food and nutrient consumption, food grouping problems, and in the relative risks included to estimate the health impacts resulting from changes in food consumption. Here we considered whether uncertainty around the changes in food consumption, health, and burden of disease were considered, and whether uncertainty values (such as a 95% uncertainty intervals) were reported.

**Assessment of quality features of PEs and epidemiological components of simulation studies**

|  | **PEs (PE)** | | | | | | | **Epidemiological model** | | | | |
| --- | --- | --- | --- | --- | --- | --- | --- | --- | --- | --- | --- | --- |
| **First Author (year), Country** | **Complete food demand system**  **🗸/🗴 (food categories)***** | **Input data short – or long-run*** | **Own- and cross-PEs**  **🗸/🗴** | **Differential PEs by socio-economic group**  **🗸/🗴** | **Source of PE valid and appropriate**  **🗸/🗴** | | **Uncertainty of PE estimate included in overall model uncertainty analsyes**  **🗸/🗴** | **Source of consumption, prevalence, and mortality data valid and appropriate**  **🗸/🗴** | | **Consumption data collected/projected over time** | **Model validated**  **🗸/🗴** | **Uncertainty of model addressed with deterministic or probabilistic sensitivity analyses**  **🗸/🗴** |
| **Allais (2010), France [**[**4**](#_ENREF_4)**]** | **🗸 (22 food categories)** | **Long-run** | **🗸** | **🗸** | **Relevant country**  **SE’s reported**  **Low random error of estimates**  **Sufficient variation in price** | **🗸**  **🗸**  **🗴**  **🗸** | **🗴** | **Relevant country**  **Sufficient size Representative** | **🗸**  **🗸**  **🗸** | **🗸**  **Panel data** | **🗴** | **🗴** |
| Andreyeva (2011) U.S.[[5](#_ENREF_5)] | 🗴 (1 food category) | Long-run | 🗴 | 🗴 | Relevant country  SE’s reported  Low random error of estimates  Sufficient variation in price | 🗸  🗸  🗸  🗸 | 🗴 | Relevant country  Sufficient size Representative | 🗸  🗸  🗸 | 🗸  Aggregate sales data | 🗴 | 🗴 |
| Bahl (2003) Ireland [[6](#_ENREF_6)] | 🗸 (2 food categories) | Long-run | 🗸 | 🗴 | Relevant country**  SE’s reported  Low random error of estimates  Sufficient variation in price | 🗸  🗴  🗴  🗸 | 🗴 | Natural experiment****** |  | 🗸  Time series | 🗴 | 🗴 |
| Cash (2005), U.S. [[7](#_ENREF_7)] | 🗴 (3 food categories) | Short-run | 🗴 | 🗸 | Relevant country  SE’s reported  Low random error of estimates  Sufficient variation in price | 🗸  🗸  🗸  🗸 | 🗸 | Relevant country  Sufficient size Representative | 🗸  🗸  🗸 | 🗸  Cross-sectional (two time points) | 🗴 | 🗸 |
| Chaloupka (2011) U.S. (Illinois) [[8](#_ENREF_8)] | 🗴 (2 food categories) | Long-run | 🗴 | 🗴 | Relevant country**  SE’s reported  Low random error of estimates  Sufficient variation in price | 🗸  🗸  🗸  🗸 | 🗴 | Natural experiment****** |  | 🗸  Aggregate sales data | 🗴 | 🗴 |
| Chouinard (2007), U.S.[[9](#_ENREF_9)] | 🗴 (14 food categories) | Long-run | 🗸 | 🗸 | Relevant country  SE’s reported  Low random error of estimates  Sufficient variation in price | 🗸  🗴  🗴  🗸 | 🗴 | Relevant country  Sufficient size Representative | 🗸  🗸  🗸 | 🗸  Panel data | 🗴 | 🗴 |
| **Clarke (2010) U.K. (Jersey) [**[**10**](#_ENREF_10)**]** | **🗸 (18 food categories)** | **Long-run** | **🗸** | **🗴** | **Relevant country**  **SE’s reported**  **Low random error of estimates**  **Sufficient variation in price** | **🗸**  **🗸**  **🗸**  **🗸** | **🗴** | **Relevant country**  **Sufficient size Representative** | **🗸**  **🗸**  **🗸** | **🗴**  **Cross sectional** | **🗴** | **🗸** |
| Dharmasena (2011), U.S. [[11](#_ENREF_11)] | 🗴 (10 food categories) | Long-run | 🗸 | 🗴 | Relevant country  SE’s reported  Low random error of estimates  Sufficient variation in price | 🗸  🗴  🗴  🗸 | 🗴 | Relevant country  Sufficient size Representative | 🗸  🗸  🗸 | 🗸  Panel data | 🗴 | 🗴 |
| Dong (2009), U.S. [[12](#_ENREF_12)] | 🗴 (2 food categories) | Short-run | 🗴 | 🗸 | Relevant country  SE’s reported  Low random error of estimates  Sufficient variation in price | 🗸  🗴  🗴  🗴 | 🗴 | Relevant country  Sufficient size Representative | 🗸  🗸  🗸 | 🗸  Cross-sectional (four time points) | 🗴 | 🗸 |
| Fantuzzi (2008) U.S. [[13](#_ENREF_13),[14](#_ENREF_14)] | 🗴 (26 food categories) | Long-run | 🗸 | 🗴 | Relevant country  SE’s reported  Low random error of estimates  Sufficient variation in price | 🗸  🗴  🗴  🗸 | 🗴 | Relevant country  Sufficient size Representative | 🗸  🗸  ~ | 🗸  Aggregate sales data | 🗴 | 🗴 |
| Finkelstein (2010), U.S. [[15](#_ENREF_15)] | 🗴 (7 food categories) | Short-run | 🗸 | 🗴 | Relevant country  SE’s reported  Low random error of estimates  Sufficient variation in price | 🗸  🗴  🗴  🗸 | 🗴 | Relevant country  Sufficient size Representative | 🗸  🗸  🗸 | 🗸  Panel data | 🗴 | 🗴 |
| Fletcher (2008), U.S. [[16](#_ENREF_16)] | 🗸 (2 food categories) | Long-run | 🗴 | 🗸 | Relevant country **  Low random error of estimates  Sufficient variation in price | 🗸  🗴  🗸 | 🗴 | Natural experiment****** |  | 🗸  Cross-sectional (16 time points) | 🗴 | 🗸 |
| Gabe (2008), U.S. [[17](#_ENREF_17)] | 🗴 (3 food categories) | Long-run | 🗴 | 🗴 | Relevant country **  SE’s reported  Low random error of estimates  Sufficient variation in price | 🗸  🗴  🗴  🗸 | 🗴 | Natural experiment****** |  | 🗸  Aggregate sales data | 🗴 | 🗴 |
| Gelbach (2007) U.S. [[18](#_ENREF_18)] | 🗸 (3 food categories) | Long-run | 🗴 | 🗴 | Relevant country  SE’s reported  Low random error of estimates  Sufficient variation in price | 🗸  🗴  🗴  🗸 | 🗴 | Relevant country  Sufficient size Representative | 🗸  🗸  🗸 | 🗸  Aggregate sales data | 🗴 | 🗴 |
| Gustavsen (2005), Norway [[19](#_ENREF_19)] | 🗸 (4 food categories) | Long-run | 🗸 | 🗴 | Relevant country  SE’s reported  Low random error of estimates  Sufficient variation in price | 🗸  🗴  🗴  🗸 | 🗴 | Relevant country  Sufficient size Representative | 🗸  🗸  🗸 | 🗸  Cross-sectional (10 time points) | 🗴 | 🗴 |
| **Jensen (2007) Denmark [**[**20**](#_ENREF_20)**]** | **🗸 (16 food categories)** | **Long-run** | **🗸** | **🗴** | **Relevant country**  **SE’s reported**  **Low random error of estimates**  **Sufficient variation in price** | **🗸**  **🗴**  **🗴**  **🗸** | **🗴** | **Relevant country**  **Sufficient size Representative** | **🗸**  **🗸**  **🗸** | **🗸**  **Aggregate sales data** | **🗴** | **🗴** |
| Kotakorpi (2011) | 🗸 (6 food categories) | Long-run | 🗸 | 🗸 | Relevant country  SE’s reported  Low random error of estimates  Sufficient variation in price | 🗸  🗸  🗸  🗸 | ~ | Relevant country  Sufficient size Representative | 🗸  🗸  🗸 | 🗸  Cross-sectional (4 time points) | 🗴 | 🗴 |
| Kuchler (2005), U.S.  [[21](#_ENREF_21)] | 🗴 (3 food categories) | Short-run | 🗸 | 🗸 | Relevant country  SE’s reported  Low random error of estimates  Sufficient variation in price | 🗸  🗴  🗴  🗸 | 🗴 | Relevant country  Sufficient size Representative | 🗸  🗸  🗸 | 🗸  Panel data | 🗴 | 🗴 |
| LaCroix (2010), France [[22](#_ENREF_22)] | 🗸 (four food categories) | Short-run | 🗸 | 🗸 | Relevant country  SE’s reported  Low random error of estimates  Sufficient variation in price | 🗸  🗴  🗴  🗴 | 🗴 | Relevant country  Sufficient size Representative | 🗸  🗴  🗴 | 🗴 Cross-sectional | 🗴 | 🗴 |
| Marshall (2000), U.K. [[23](#_ENREF_23)] | 🗴 (6 food categories) | Long-run | 🗴 | 🗴 | Relevant country  SE’s reported  Low random error of estimates  Sufficient variation in price | 🗴  🗴🗴  🗴 | 🗴 | Relevant country  Sufficient size Representative | 🗸  🗸  🗸 | 🗴 Unclear | 🗴 | 🗴 |
| **Mytton (2007), U.K. [**[**24**](#_ENREF_24)**]** | **🗸 (18 food categories)** | **Long-run** | **🗸** | **🗴** | **Relevant country**  **SE’s reported**  **Low random error of estimates**  **Sufficient variation in price** | **🗸**  **🗸**  **🗸**  **🗸** | **🗴** | **Relevant country**  **Sufficient size Representative** | **🗸**  **🗸**  **🗸** | **🗴 Cross-sectional** | **🗴** | **🗸** |
| **Nnoaham (2009), U.K. [**[**25**](#_ENREF_25)**]** | **🗸 (18 food categories)** | **Long-run** | **🗸** | **🗴** | **Relevant country**  **SE’s reported**  **Low random error of estimates**  **Sufficient variation in price** | **🗸**  **🗸**  **🗸**  **🗸** | **🗴** | **Relevant country**  **Sufficient size Representative** | **🗸**  **🗸**  **🗸** | **🗴 Cross-sectional** | **🗴** | **🗸** |
| Nordstrom (2007), Sweden [[26](#_ENREF_26),[27](#_ENREF_27)] | 🗴 (8 food categories) | Short-run | 🗴 | 🗸 | Relevant country  SE’s reported  Low random error of estimates  Sufficient variation in price | 🗸  🗴  🗴  🗸 | 🗴 | Relevant country  Sufficient size Representative | 🗸  🗸  🗸 | 🗸  Aggregate sales data | 🗴 | 🗴 |
| **Oaks (2005), U.S. [**[**28**](#_ENREF_28)**]** | **🗸 (all food groups)** | **Long-run** | **🗸** | **~** | **Relevant country****  **SE’s reported**  **Low random error of estimates**  **Sufficient variation in price** | **🗸**  **🗴**  **🗸**  **🗸** | **🗴** | **Natural experiment**** |  | 🗸  **Aggregate sales data** | **🗴** | **🗴** |
| Sacks (2010), Australia [[29](#_ENREF_29)] | 🗴 (9 food categories) | Long-run | 🗸 | 🗴 | Relevant country  SE’s reported  Low random error of estimates  Sufficient variation in price | 🗴  🗴🗸  🗸 | 🗴 | Relevant country  Sufficient size Representative | 🗸  🗸  🗸 | 🗴 Cross sectional | 🗴 | 🗴 |
| Sassi (2009) U.S. [[30](#_ENREF_30)] | 🗴 (2 food categories) | Long-run | 🗸 | 🗴 | Relevant country  SE’s reported  Low random error of estimates  Sufficient variation in price | 🗴  🗸🗸  🗸 | 🗴 | Relevant country  Sufficient size Representative | 🗸  🗸  🗸 | 🗸  Cross sectional (several time points) | 🗴 | 🗸 |
| Schroeter (2008), U.S.  [[31](#_ENREF_31)] | 🗸 (2 food categories) | Not a standard demand model | 🗸 | 🗴 | Relevant country  SE’s reported  Low random error of estimates  Sufficient variation in price | 🗸  🗴  🗴  🗴 | 🗴 | Relevant country  Sufficient size Representative | 🗸  🗸  🗸 | 🗸  Cross sectional (nine time points) | 🗴 | 🗴 |
| **Smed (2007), Denmark [**[**32**](#_ENREF_32)**]** | **🗸 (23 food categories)** | **Long-run** | **🗸** | **🗸** | **Relevant country**  **SE’s reported**  **Low random error of estimates**  **Sufficient variation in price** | **🗸🗸🗸**  **🗸** | **🗴** | **Relevant country**  **Sufficient size Representative** | **🗸**  **🗸**  **🗸** | **🗸**  **Cross sectional (two time points)** | **🗴** | **🗴** |
| Smith (2010), U.S. [[33](#_ENREF_33)] | 🗴 (8 food categories) | Long-run | 🗸 | 🗴 | Relevant country  SE’s reported  Low random error of estimates  Sufficient variation in price | 🗸  🗸  🗸  🗸 | 🗴 | Relevant country  Sufficient size Representative | 🗸  🗸  🗸 | 🗸  Panel data | 🗴 | 🗴 |
| Tefft (2008), U.S. [[34](#_ENREF_34)] | 🗴 (2 food categories) | Long-run | 🗴 | 🗴 | Relevant country  SE’s reported  Low random error of estimates  Sufficient variation in price | 🗸  🗸  🗸  🗸 | 🗴 | Relevant country  Sufficient size Representative | 🗸  🗸  🗸 | 🗸  Cross sectional (12 time points) | 🗴 | 🗴 |
| Tiffin (2011), U.K. [[35](#_ENREF_35),[36](#_ENREF_36)] | 🗸 (7 food categories) | Short-run | 🗸 | 🗴 | Relevant country  SE’s reported  Low random error of estimates  Sufficient variation in price | 🗸🗸🗸  🗴 | 🗴 | Relevant country  Sufficient size Representative | 🗸  🗸  🗸 | 🗴 Cross sectional | 🗴 | 🗴 |
| Zhen (2010), U.S. [[37](#_ENREF_37)] | 🗴 (9 food categories) | Long-run and short-run | 🗸 | 🗸 | Relevant country  SE’s reported  Low random error of estimates  Sufficient variation in price | 🗸  🗴  🗴  🗸 | 🗴 | Relevant country  Sufficient size Representative | 🗸  🗴  🗴 | 🗸  Panel data | 🗴 | 🗴 |

**Bolded studies** considered high quality

Short-run PEs are derived from short-run demand equations, and long-run PEs are derived from long-run demand equations. The former is a dynamic model including the rate of change of food purchases and assumes consumers adjust immediately to price changes. The latter is a static model allowing for habit formation of the consumer over time in response to price changes. Long-run PEs are generally more elastic compared with short-run PEs[[38](#_ENREF_38)]

** Natural case study

*** Complete demand system did not have to include food consumed away from home

~Not possible to determine from report

**Characteristics of included studies**

**Methods and findings of studies assessing the effects of pricing strategies on diet (food/nutrient intake/purchases)**

| **First Author (year), Country** | **Model food groups** | **Interventions modelled** | **Dataset** | **Outcomes** | **Impact** | **Pragmatic issues addressed** |
| --- | --- | --- | --- | --- | --- | --- |
| **Impact on food or nutrient consumption** | | | | | | |
| **Studies assessing taxes** | | | | | | |
| Allais (2010), France [[4](#_ENREF_4)] | *Model:* Complete demand system including 22 food groups | *Taxes:*  10% on foods high in fat and sugar: cheese/ butter/cream, sugar-fat products, and pre-prepared meals | *Dataset:* TNS Worldpanel data 1996 to 2001 (4 weeks per household)  *N:* ~5,000  *Socioeconomic group:* effects assessed for modest and well-off groups separately. | *Total fat and sugar tax:*  Modest and well-off:  Energy, protein, sugar, total fat, saturated fat, and sugar purchases from all taxed products for both income groups (% change)  Alcohol purchases for both income groups (% change)  Greater impacts for modest compared with well-off. | -  + | *Taxes regressive:* Yes *Compensation buying:* NR  *Definition of healthy/less healthy foods:* n/a  *Application and size of tax:* Flat rate tax on total cost applied at point of sale |
| Andreyeva (2011) U.S.[[5](#_ENREF_5)] | *Model:* Includes only SSB | *Taxes:*  1 penny per ounce on carbonated soft drinks, fruit drinks, ready to drink teas, sports drinks, flavoured water, and ready to drink coffees | *Dataset:* Regional industry sales data for 2008 on carbonated soft drinks, fruit drinks, and ready to drink teas; and national industry sales data for 2008 on sports drinks, flavoured water, and ready to drink coffees. 2007 to 2015 Census projection data used to estimate population size.  *N:* NR  *Socioeconomic group:* NR | *SSB tax:*  Carbonated sugars sweetened beverage purchases (%)  Diet SSB purchases (%)  Fruit drink purchases (%)  Sports drink purchases (%)  Regular ready to drink tea purchases (%)  Diet ready to drink tea purchases (%)  Flavoured water purchases (%)  Energy drink purchases (%)  Ready to drink coffee purchases (%)  Total SSB purchases (%)  (-6% to -27% decreases) | -  0  -  -  -  0  -  -  -  - | *Taxes regressive:* NR  *Compensation buying:* NR  *Definition of healthy/less healthy foods:* n/a  *Application and size of tax:* Rate per volume tax applied at point of sale |
| Gabe  oupka (2011), U.S. (Illinois)[[8](#_ENREF_8)] | *Model:* Includes 2 types SSBs: all beverages and SSB only | *Taxes:*  One cent per ounce on SSBs; one cent per ounce on SSB and diet versions; two cent per ounce on SSB; and two cent per ounce on SSB and diet versions | *Dataset:* For beverages: Beverage Marketing Corporation sales (2009) and Beverage World sales (2009) extrapolated over period 2000 to 2009. For epidemiological model: 2009 Behavioural Risk Factor Surveillance System data for Illinois and 2007 survey of Children’s Health  *N:* NR but state sales  *Socioeconomic group:* NR | *One penny per ounce SSB:*  SSB consumption  Frequency of SSB consumption  *One penny per ounce SSB + diet:*  SSB consumption  Frequency of SSB consumption  *Two penny per ounce SSB:*  SSB consumption  Frequency of SSB consumption  *Two penny per ounce SSB + diet:*  SSB consumption  Frequency of SSB consumption | -  -  -  -  -  -  -  - | *Taxes regressive:* n/a  *Compensation buying:* Estimated using literature to be 50% of energy intake to other beverages  *Definition of healthy/less healthy foods:* n/a  *Application and size of tax:* Rate per volume applied at point of sale |
| Chouinard (2007), U.S.[[9](#_ENREF_9)] | *Model:* Includes 14 dairy products | *Taxes:*  10% and 50% flat tax on dairy products | *Dataset:* Info scanner data from 1997 to 1999 for 23 U.S. cities  *N:* ~50,000 to 10 million per city  *Socioeconomic group:* Burden of tax examined by household income. | *10% fat tax on dairy:*  Fat purchased from low fat milk, cream, ice cream and flavoured yoghurt  Fat purchased from whole milk, coffee additives, cheese, cream cheese, butter  Fat purchased from no fat milk  *50% fat tax on dairy:*  Results ~5x those of 10% tax  Similar impacts across groups therefore reported overall only | +  -  0 | *Taxes regressive:* Burden of tax falls predominantly on lowest income groups  *Compensation buying:* NR  *Definition of healthy/less healthy foods:* n/a  *Application and size of tax:* Flat tax on total cost applied at point of sale |
| Dharmasena (2011), U.S. [[11](#_ENREF_11)] | *Model:* Includes 10 categories of non-alcoholic beverages: sports drinks, regular and diet soft drinks, whole and low fat milk, fruit drinks and juices, bottled water, coffee and tea. | *Taxes:*  20% on sugar three categories of sweetened beverages: sports drinks, regular soft drinks, and fruit drinks | *Dataset:* Nielsen Homescan panel data for four regions in U.S. from 1998 to 2003  *N:* NR  *Socioeconomic group:* NR | *Selected SSB tax:*  Sports drink purchases (%)  Regular soft drink purchases (%)  Fruit drink purchases (%)  High fat milk purchases (%)  Low fat milk purchases (%)  Fruit juice purchases (%)  Bottled water purchases (%)  Coffee purchases (%)  Tea purchases (%)  (changes range from -130% for sports drinks to +28% for fruit juices)  Overall energy purchases (-450 cal / 1,890 kJ per month) | -  -  -  -  0  0  -  0  0  - | *Taxes regressive:* NR  *Compensation buying:* NR  *Definition of healthy/less healthy foods:* n/a  *Application and size of tax:* Flat rate tax applied at point of sale |
| Fantuzzi (2008) U.S. [[13](#_ENREF_13)] | *Model:* Includes 26 brands of carbonated soft drinks | *Taxes:*  20% flat rate on soft drinks, and U.S. 10c per calorie rate on soft drinks | *Dataset:* Scanner data for 20 U.S. cities  *N:* 40,000  *Socioeconomic group:* effects assessed for lower and higher income groups | *Flat rate soft drink tax:*  Energy intake (calories)  *Per nutrient soft drink tax:*  Energy intake (calories)  (very small changes <5,000 calories/year)  Positive impact on food purchases and health with greater PE’s for lower income groups. | -  - | *Taxes regressive:* Yes *Compensation buying:* NR  *Definition of healthy/less healthy foods:* n/a  *Application and size of tax:* Combination of flat rate tax on total cost and tax amount per calorie applied at point of sale |
| Finkelstein (2010), U.S. [[15](#_ENREF_15)] | *Model:* Seven categories of beverage: sugar-sweetened beverages, diet carbonated beverages, sports/energy drinks, fruit drinks, fruit juice, skim milk, and whole milk | *Taxes:*  20% and 40% on all carbonated beverages and all SSBs | *Dataset:* Nielsen Homescan panel data for 2006  *N:* 40,000  *Socioeconomic group:* effects assessed across four income groups (1=lowest). | *20% carbonated beverage tax:*  Energy from carbonated soft drinks:  All  Income groups 1 to 4  Energy from all SSBs:  All  Income groups 1 & 4  Income groups 2 & 3  *40% carbonated beverage tax:*  Energy from carbonated soft drinks:  All  Income groups 1 to 4  Energy from all SSBs:  All  Income groups 1 & 4  Income groups 2 & 3  *20% SSB tax:*  Energy from all SSBs:  All  Income groups 1 & 4  Income groups 2 & 3  Energy from carbonated soft drinks:  All  Income groups 1 to 4  *40% SSB tax:*  Energy from all SSBs:  All  Income groups 1 to 3  Income group 4  Energy from carbonated soft drinks:  All  Income groups 1 to 4  Effects of carbonated beverage tax on all beverages driven by middle income groups only | -  -  -  0  -  -  -  -  0  -  -  0  -  -  -  -  -  0  -  - | *Taxes regressive:* No as lower income households buy more expensive beverages.  *Compensation buying:* Appears people may buy other sweetened beverages when only carbonated beverages are taxed  *Definition of healthy/less healthy foods:* n/a  *Application and size of tax:* Flat rate tax on total cost applied at point of sale |
| Gabe (2008), U.S. [[17](#_ENREF_17)] | *Model:* Includes 3 types of soft drink:  Pepsi Cola, Coca cola and Powerade. | *Taxes:*  11.9% on soft drinks and 8.1% on sports drinks | *Dataset:* not specified, but obtained from U.S. government and Beverage Association  *N:* NR  *Socioeconomic group:* NR | *Soft drink tax:*  Purchases of soft drinks (% change)  *Sports drink tax:*  Purchases of sports drinks (% change)  (changes between -3 and 5%) | -  - | *Taxes regressive:* n/a  *Compensation buying:* NR  *Definition of healthy/less healthy foods:* NR  *Application and size of tax:* Flat rate subsidy on total cost applied at point of sale |
| Gustavsen (2005), Norway [[19](#_ENREF_19)] | *Model:* Four food categories: traditional vegetables, salad vegetables, industrially processed vegetables, and all other foods | *Taxes:*  10.8% price increase on soft drinks at point of sale; and 27.3% price increase on soft drinks at point of sale | *Dataset:* Household expenditure surveys for Norway 1989 to 1999  *N:* 14,000  *Socioeconomic group:* NR  Analysis by 5 quantiles of soft drink consumption (1=lowest) | *10.8% soft drink tax:*  Purchases of soft drinks (% change)  (-6 to -17% increasing by consumption quantile)  *27.3% soft drink tax:*  Purchases of soft drinks (% change)  (-17 to -44% increasing by consumption quantile) | -  - | *Taxes regressive:* NR  *Compensation buying:* NR  *Definition of healthy/less healthy foods:* n/a  *Application and size of tax:* Flat rate tax on total cost applied at point of sale (theoretically proportion of tax in 27.3% scenario applied at production, but actual price increase at point of sale modelled) |
| Jensen (2007) Denmark [[20](#_ENREF_20)] | *Model:* Includes 16 food categories and nutrients: milk, butter and fat, cheese, meat, eggs, fish, flour, sugar, fruit and vegetables, total and saturated fat, sugar, fibre | *Taxes:* 8 DKK tax per kg total fat; 14 DKK per kg saturated fat; 5.6 DKK per kg sugar; | *Dataset:* Annual purchase data Statistics Denmark 1972 - 96  *N:* population-based sales data  *Socioeconomic group:* NR | *Total fat tax:*  Milk, butter and fat, cheese, meat, total and saturated fat purchases  Eggs, fish, flour, sugar, fruit and vegetables and fibre  *Saturated fat tax:*  Milk, butter and fat, cheese, meat, total and saturated fat  Eggs, fish, flour, sugar, fruit and vegetables and fibre  *Sugar tax:*  Milk, butter and fat, cheese, eggs, meat, fish, flour, fruit and vegetables, total and saturated fat, and fibre  Sugar | -  +  -  +  +  - | *Taxes regressive: NR*  *Compensation buying:* NR  *Definition of healthy/less healthy foods:* n/a  *Application and size of tax:* Rate per amount of nutrient applied at point of sale |
| Kotakorpi (2011) Finland [[39](#_ENREF_39)] | *Model:* Includes 6 food categories: bread, meat, fish, fruit and vegetables, sugar and sweets, and other | *Taxes:* 1€ per kg added sugar | *Dataset:* Finish Household Budget Surveys 1995, 1998, 2001, & 2006 for demand models and Health Survey 2000 for change in food intake and nutrients  *N:* Household budget surveys  ~17,000 households; Health Survey =10,000 individuals  *Socioeconomic group:* three income groups based on disposable income (low,med,high). | *Sugar tax:*  All income groups:  Bread (Sig)  Meat  Fish  Fruit and vegetables  Sugar and sweets  (Sig = p<0.05)  Higher elasticities for low income groups therefore health impacts greater and overall inequalities decreased. | -(Sig)  +(Sig)  +  -  -(Sig) | *Taxes regressive:* Yes, mildly.  *Compensation buying:* Yes, with small but insignificant decrease in fruit and vegetables  *Application and size of tax:* Rate per amount of nutrient applied at point of sale |
| Kuchler (2005), U.S.  [[21](#_ENREF_21)] | *Model:* Includes 3 categories of snack foods: potato chips, all chips, and salty snacks | *Taxes:*  1%, 10% and 20% taxes on potato chips, all chips, and salty snacks | *Dataset:* AC Nielsen homescan panel sales data 1999  *N:* 12,000 households in panel for ≥ 10 months  *Socioeconomic group:* NR | *1%, 10% and 20% potato chip taxes:*  Potato chips  All chips  All salty snacks  (very small changes)  *1%, 10% and 20% all chips taxes:*  Potato chips  All chips  All salty snacks  (very small changes)  *1%, 10% and 20% all salty snacks taxes:*  Potato chips  All chips  All salty snacks  (very small changes) | -  -  -  -  -  -  -  -  - | *Taxes regressive:* NR  *Compensation buying:* NR  *Definition of healthy/less healthy foods:* n/a  *Application and size of tax:* Flat tax on total cost applied at point of sale |
| Marshall (2000), U.K. [[23](#_ENREF_23)] | *Model:* Includes 6 categories of food contributing to saturated fat in British diet: whole milk, cheese, butter, biscuits, buns cakes and pastries, puddings, and ice cream | *Taxes:*  17.5% on 6 categories of food contributing to saturated fat in British diets | *Dataset:* Dietary intake data only used from 1990 survey of British adults  *N:* NR  *Socioeconomic group:* NR | *17.5% taxes on foods contributing to saturated fat intakes:*  Whole milk  Cheese  Butter  Biscuits  Buns, cakes, and pastries  Puddings and ice cream  Total  (% reductions all <0.5%) | -  -  -  -  -  -  - | *Taxes regressive:* no calculations undertaken but suggested saturated fat tax likely to be regressive as low income spend higher proportion on food  *Compensation buying:* NR  *Definition of healthy/less healthy foods:* food contributing substantially to saturated fat intakes in Britain  *Application and size of tax:* Flat tax on total cost applied at point of sale |
| Mytton (2007), U.K. [[24](#_ENREF_24)] | *Model:* Includes 18 major food categories | *Taxes:*  17.5% on principle sources of saturated fat; less healthy foods; and combination of taxes to achieve best health outcome for lowest cost to consumer | *Dataset:* National Food Survey of Great Britain 2000  *N:* NR  *Socioeconomic group:* NR | *Saturated fat tax:*  Saturated fat intake (% change)  Salt intake (% change)  Non-milk extrinsic sugar intake (% change)  Energy (% change)  Fruit and vegetable intake (% change)  *Less healthy food tax:*  Saturated fat intake (% change)  Salt intake (% change)  Non-milk extrinsic sugar intake (% change)  Energy (% change)  Fruit and vegetable intake (% change)  *Best outcome tax:*  Saturated fat intake (% change)  Salt intake (% change)  Non-milk extrinsic sugar intake (% change)  Energy (% change)  Fruit and vegetable intake (% change)  (all changes were small) | -  +  -  +  -  +  -  -  -  -  +  -  -  -  - | *Taxes regressive:* NR  *Compensation buying:* effect on 5 nutrients and one food group reported  *Definition of healthy/less healthy foods:* less healthy foods defined using SSCg3d [[40](#_ENREF_40)] nutrient profiling model  *Application and size of tax:* Flat tax on total cost applied at point of sale |
| Nnoaham (2009), U.K. [[25](#_ENREF_25)] | *Model:* Includes 18 major food categories | *Taxes:*  17.5% on principle sources of saturated fat; less healthy foods; and combination of taxes to achieve best health outcome for lowest cost to consumer | *Dataset:* Expenditure and Food Survey 2003 to 2006  *N:* NR  *Socioeconomic group:* effects assessed across 5 categories of household income (1=lowest). | *Saturated fat tax:*  Energy intake (% change):  Overall  Income quintiles 1 to 5  Saturated fat intake (% change):  Overall  Income quintiles 1 to 5  Salt intake (% change):  Overall  Income quintiles 1 to 5  Fruit and vegetable intake (% change):  Overall  Income quintiles 1 to 5  *Less healthy food tax:*  Energy intake (% change):  Overall  Income quintiles 1 to 5  Saturated fat intake (% change):  Overall  Income quintiles 1 to 5  Salt intake (% change):  Overall  Income quintiles 1 to 5  Fruit and vegetable intake (% change):  Overall  Income quintiles 1 to 5  Similar impacts across income groups. | -  -  -  -  +  +  +  +  -  -  -  -  -  -  -  - | *Taxes regressive:* Yes  *Compensation buying:* NR  *Definition of healthy/less healthy foods:* less healthy foods defined using SSCg3d [[40](#_ENREF_40)] nutrient profiling model  *Application and size of tax:* Flat tax on total cost applied at point of sale |
| Sacks (2010), Australia [[29](#_ENREF_29)] | *Model: Model:* Includes 9 food categories: regular bread and rolls, cereal-based products and dishes, cheese, muscle meat, poultry and other feathered game, sausages frankfurters and saveloys, snack foods, confectionery, soft drinks flavoured mineral waters and electrolyte drinks | *Taxes:*  10% on ‘junk food’ | *Dataset:* National Nutrition Survey data 1995  *N:* NR  *Socioeconomic group:* NR | *Junk (less healthy) food tax:*  Food purchases and energy intake (kJ/day) for males and females:  Cereal based products and dishes, sausages frankfurters and saveloys, snack foods, confectionary, soft drinks flavoured water and electrolyte drinks  Regular bread and rolls, cheese, muscle meal, poultry and other game  Total  (~-175 and 120 kJ/day for males and females energy intake, respectively) | -  +  - | *Taxes regressive:* NR  *Compensation buying:* NR  *Definition of healthy/less healthy foods:* less healthy foods selected based on non-core foods high in saturated fat, sugar, and/or salt  *Application and size of tax:* Flat rate tax applied at point of sale |
| Smed (2007), Denmark [[32](#_ENREF_32)] | *Model:* Includes 23 food groups | *Taxes:* 5% tax on meat, butter and cheese; 7.9 DKK tax per kg saturated fat; 10 DKK per kg tax on sugar | *Dataset:* Gfk consumer scan data (1997 to 2000)  *N:* ~2,000  *Socioeconomic group:* impact of combined interventions assessed across 5 social classes (1 = lowest). | *Meat, butter, fat tax:*  Energy:  Social class 1&2 (high)  Social classes 3-5  Saturated fat:  Social class 1&2 (high)  Social classes 3-5  Sugar:  All social classes  *Saturated fat tax:*  Energy:  Social class 1&2 (high)  Social classes 3-5  Saturated fat:  Social class 1&2 (high)  Social classes 3-5  Sugar:  Social class 1&2 (high)  Social classes 3-5  *Sugar tax:*  Energy:  Social class 1&2 (high)  Social classes 3&4  Social class 5  Saturated fat:  Social class 1&2 (high)  Social classes 3-5  Sugar:  All social classes  Varied impact on food purchases by social class. | +  -  +  -  +  +  -  +  -  -  +  +  -  +  -  +  - | *Taxes regressive:* Yes  *Compensation buying:* NR  *Definition of healthy/less healthy foods:* n/a  *Application and size of tax:* Combination of flat taxes and rate per amount of nutrient applied at point of sale |
| Smith (2010), U.S. [[33](#_ENREF_33)] | *Model:* Includes 8 categories of soft drinks based on energy content: caloric sweetened beverages, diet drinks, skim milk, low fat milk, 100% fruit and vege juices, coffee/tea, and bottled water | *Taxes:*  20% on caloric sweetened beverages | *Dataset:* Nielsen Homescan household scanner data 1998 to 2007 and National Health and Examination Survey data 2003 to 2006  *N:* NR  *Socioeconomic group:* NR | *Caloric sweetened beverage tax:*  Energy intake from caloric sweetened beverages:  Adults  Children  (~ 25% larger for children)  Energy intake from all beverages:  Adults  Children  (~ 300% larger for children) | -  -  -  - | *Taxes regressive:* NR  *Compensation buying:* NR outside of beverages  *Definition of healthy/less healthy foods:* n/a  *Application and size of tax:* Flat rate tax applied at point of sale |
| Tefft (2008), U.S. [[34](#_ENREF_34)] | *Model:* Includes soft drinks and snack foods | *Taxes:*  Variation in sales taxes across states used to estimate tax increase of 10% on soft drinks | *Dataset:* Consumer Expenditure Diary Survey 1990 to 2002  *N:* 82,175  *Socioeconomic group:* effects assessed separately for Black and Hispanic and effects of income assessed. | *Soft drink tax:*  Household soft drink purchases  (change very small < 1%)  Snack food purchases  Socioeconomic groups were not more or less affected by tax so overall findings reported. | -  - | *Taxes regressive: NR*  *Compensation buying:* NR  *Definition of healthy/less healthy foods:* n/a  *Application and size of tax:* Flat rate tax on total cost applied at point of sale |
| Zhen (2010), U.S. [[37](#_ENREF_37)] | *Model:* Includes 9 beverage categories: regular and diet carbonated soft drinks, whole and low fat milk, bottled water, sports and energy drinks, fruit juice, coffee, tea, and sugar-sweetened fruit drinks | *Taxes:*  1/2 cent per ounce on carbonated soft drinks, sports and energy drinks, and sugar sweetened juice drinks | *Dataset:* Nielsen Homescan household scanner data 2004 to 2006  *N:* 150 synthetic households developed (75 each for low and high income)  *Socioeconomic group:* effects assessed separately for low and high income groups (low income = below 185% of poverty line) | *SSB tax:*  Low income purchases over long term:  Regular and diet carbonated soft drinks, whole milk, bottled water, sports and energy drinks, and sugar sweetened fruit drinks  Low fat milk, fruit juice, tea and coffee  High income purchases over long term:  Regular and diet carbonated soft drinks and sugar sweetened fruit drinks  Whole and low fat milk, bottled water, sports and energy drinks, fruit juice, coffee, and tea  Health benefits may be better for low income | -  +  -  + | *Taxes regressive:* Yes *Compensation buying:* NR outside of beverages  *Definition of healthy/less healthy foods:* n/a  *Application and size of tax:* Rate per volume tax applied at point of sale |
| **Studies assessing subsidies** | | | | | | |
| Bahl (2003) Ireland [[6](#_ENREF_6)] | *Model:* Ecological based on introduction and subsequent removal of tax on soft drinks in Ireland. Includes two categories only: soft drinks and other foods | *Subsidies:* Tax decreased from IR 0.37/gallon to IR 0.29/gallon | *Dataset:* Sales of soft drinks from Government of Ireland Reports  *N:* population-based sales data  *Socioeconomic group:* NR | *Soft drink subsidy:*  Purchases of soft drinks (%) | + | *Compensation buying: NR*  *Definition of healthy/less healthy foods:* all soft drinks  *Application and size of subsidy:* Subsidy per gallon in tax at point of production |
| LaCroix (2010) France [[22](#_ENREF_22)] | *Model:* Includes all foods categorised into: Fruit and vegetables, healthy products, neutral products, and unhealthy products | *Subsidies:* 30% on fruit and vegetables | *Dataset:* Experimental data used from laboratory study *N:* 107 women  *Socioeconomic group:* 74 women were low income; 33 others used as reference group. | *Fruit and vegetable subsidy:*  Fruit and vegetable purchases:  Low income  Reference group  Healthy product purchases:  Low income  Reference group  Unhealthy product purchases:  Low income  Reference group  Neutral product purchases:  Low income  Reference group  Varied impact on food purchases for low income. Similar impact for low and high income groups | +  +  _  +  _  _  _  _ | *Compensation buying:* Higher income increase purchase of other healthy foods and lower income increase purchases of unhealthy products.  *Definition of healthy/less healthy foods:* French Food Standards Agency Nutrient Profiling Model  *Application and size of subsidy:* Flat rate subsidy on total cost applied at point of sale. Lower income decrease their budget spent. |
| Dong (2009), U.S. [[12](#_ENREF_12)] | *Model:* Includes ~two food categories: fruit and vegetables | *Subsidies:*  10% on fruit and vegetables | *Dataset:* 2004 Nielsen homescan data  *N:* NR  *Socioeconomic group:* Low income (below 130% of poverty threshold) and all U.S. | *Fruit and vegetable subsidy:*  Fruit and vegetable purchases (cups)  (small changes 2 to 5%) | + | *Compensation buying:* NR  *Definition of healthy/less healthy foods:* NR  *Application and size of subsidy:* Flat rate subsidy on total cost applied at point of sale |
| Gustavsen (2005), Norway [[19](#_ENREF_19)] | *Model:* Four food categories: traditional vegetables, salad vegetables, industrially processed vegetables, and all other foods | *Subsidies:*  21.7% subsidy on soft drinks | *Dataset:* Household expenditure surveys for Norway 1989 to 1999  *N:* 14,000  *Socioeconomic group:* NR  Analysis by 5 quantiles of soft drink consumption (1=lowest) | *21.7% soft drink subsidy*  (=13 to 35% increasing by consumption quantile) | + | *Compensation buying:* NR  *Definition of healthy/less healthy foods:* n/a  *Application and size of subsidy:* Flat rate tax on total cost applied at point of sale (theoretically proportion of tax in 27.3% scenario applied at production, but actual price increase at point of sale modelled) |
| Jensen (2007) Denmark [[20](#_ENREF_20)] | *Model:* Includes 16 food categories and nutrients: milk, butter and fat, cheese, meat, eggs, fish, flour, sugar, fruit and vegetables, total and saturated fat, sugar, fibre | *Subsidies:*  12.5% subsidy on fruit and vegetables;  76.4 DKK subsidy per kg fibre | *Dataset:* Annual purchase data Statistics Denmark 1972 - 96  *N:* population-based sales data  *Socioeconomic group:* NR | *Fruit and vegetable subsidy:*  Fruit and vegetable, flour, fibre purchases  Milk, butter and fat, cheese, eggs, fish, total and saturated fat purchases  Meat purchases  *Fibre subsidy:*  Flour, fruit and vegetables, fibre purchases  Milk, butter and fat, cheese, eggs, fish, sugar, total and saturated fat purchases | +  -  0  +  - | *Compensation buying:* NR  *Definition of healthy/less healthy foods:* n/a  *Application and size of subsidy:* Rate per amount of nutrient applied at point of sale |
| Kotakorpi (2011) Finland [[39](#_ENREF_39)] | *Model:* Includes 6 food categories: bread, meat, fish, fruit and vegetables, sugar and sweets, and other | *Subsidies:* 13% removal of VAT on fresh fruit, vegetables, and fish | *Dataset:* Finish Household Budget Surveys 1995, 1998, 2001, & 2006 for demand models and Health Survey 2000 for change in food intake and nutrients  *N:* Household budget surveys  ~17,000 households; Health Survey =10,000 individuals  *Socioeconomic group:* three income groups based on disposable income (low,med,high). | *Fresh fruit and vegetable and fish subsidy:*  All income groups:  Bread (Sig)  Meat  Fish  Fruit and vegetables  Sugar and sweets  (Sig = p<0.05)  Higher elasticities for low income groups therefore health impacts greater and overall inequalities decreased | -  +(Sig)  + (Sig)  +(Sig)  - | *Compensation buying:* Yes, but with only potential effect detrimental to health being increased meat intake  *Application and size of tax:* Flat rate applied at point of sale |
| Smed (2007), Denmark [[32](#_ENREF_32)] | *Model:* Includes 23 food groups | *Subsidies:*  2% subsidy on fruit and vegetables, potatoes, and grains (fibre); 18DKK subsidy per kg fibre | *Dataset:* Gfk consumer scan data (1997 to 2000)  *N:* ~2,000  *Socioeconomic group:* impact of combined interventions assessed across 5 social classes (1 = lowest). | *Fruit, vegetable, potato, grain subsidy:*  Energy:  All social classes  Saturated fat:  All social classes  Sugar:  Social classes 1&2  Social class 3  Social classes 4&5  Varied impact on food purchases by social class. | +  -  -  0  + | *Compensation buying:* NR  *Definition of healthy/less healthy foods:* n/a  *Application and size of subsidy:* Combination of flat taxes and rate per amount of nutrient applied at point of sale |
| **Studies assessing tax and subsidy combinations** | | | | | | |
| Jensen (2007) Denmark [[20](#_ENREF_20)] | *Model:* Includes 16 food categories and nutrients: milk, butter and fat, cheese, meat, eggs, fish, flour, sugar, fruit and vegetables, total and saturated fat, sugar, fibre | *Combinations:* Tax on saturated fat and sugar and subsidy on fibre; tax on total fat and sugar and subsidy on fruit and vegetables | *Dataset:* Annual purchase data Statistics Denmark 1972 - 96  *N:* population-based sales data  *Socioeconomic group:* NR | *Saturated fat and sugar tax & fibre subsidy:*  Milk, butter and fat, cheese, meat, fish, sugar, total and saturated fat  Eggs, flour, fruit and vegetables and fibre  *Total fat and sugar tax & fruit and vegetable subsidy:*  Milk, butter and fat, cheese, eggs, meat, fish, sugar, total and saturated fat  Flour, fruit and vegetables and fibre | -  +  -  + | *Taxes regressive: NR*  *Compensation buying:* NR  *Definition of healthy/less healthy foods:* n/a  *Application and size of tax/subsidy:* Rate per amount of nutrient applied at point of sale |
| Kotakorpi (2011) Finland [[39](#_ENREF_39)] | *Model:* Includes 6 food categories: bread, meat, fish, fruit and vegetables, sugar and sweets, and other | *Combinations:* 1€ per kg added sugar and 13% removal of VAT on fresh fruit, vegetables, and fish | *Dataset:* Finish Household Budget Surveys 1995, 1998, 2001, & 2006 for demand models and Health Survey 2000 for change in food intake and nutrients  *N:* Household budget surveys  ~17,000 households; Health Survey =10,000 individuals  *Socioeconomic group:* three income groups based on disposable income (low,med,high). | *Sugar tax and fresh fruit and vegetable and fish subsidy:*  All income groups:  Bread (Sig)  Meat  Fish  Fruit and vegetables  Sugar and sweets  (Sig = p<0.05)  Higher elasticities for low income groups therefore health impacts greater and overall inequalities decreased | - (Sig)  +(Sig)  + (Sig)  +(Sig)  - (Sig) | *Taxes regressive:* Yes, mildly.  *Compensation buying:* Yes- potential effect detrimental to health being increased meat intake  *Application and size of tax/subsidy:* Rate per amount of nutrient applied at point of sale for tax and flat rate applied at point of sale for subsidy |
| LaCroix (2010) France [[22](#_ENREF_22)] | *Model:* Includes all foods categorised into: Fruit and vegetables, healthy products, neutral products, and unhealthy products | *Combinations:* 30% tax on unhealthy products combined with 30% subsidy on fruit and vegetables and other healthy products | *Dataset:* Experimental data used from laboratory study *N:* 107 women  *Socioeconomic group:* 74 women were low income; 33 others used as reference group. | *Less healthy tax + healthy subsidy:*  Low income:  Fruit and vegetable purchases  Healthy product purchases  Unhealthy product purchases  Neutral product purchases  Reference group:  Fruit and vegetable purchases  Healthy product purchases  Unhealthy product purchases  Neutral product purchases  Similar increase in purchases for low income and reference, but does not reduce inequality. | +  -  -  -  +  +  -  - | *Taxes regressive:* Yes  *Compensation buying:* Higher income increase purchases of other healthy foods and lower income decrease purchases of all other products.  *Definition of healthy/less healthy foods:* French Food Standards Agency Nutrient Profiling Model  *Application and size of tax/subsidy:* Flat rate subsidy on total cost applied at point of sale Lower income decrease their budget spent. |
| Nnoaham (2009), U.K. [[25](#_ENREF_25)] | *Model:* Includes 18 major food categories | *Combinations:* 17.5% tax on less healthy foods combined with subsidy on fruit and vegetables | *Dataset:* Expenditure and Food Survey 2003 to 2006  *N:* NR  *Socioeconomic group:* effects assessed across 5 categories of household income (1=lowest). | *Less healthy tax + fruit and vegetable subsidy:*  Energy intake (% change)  Overall  Income quintiles 1 to 5  Saturated fat intake (% change)  Overall  Income quintiles 1 to 5  Salt intake (% change)  Overall  Income quintiles 1 to 5  Fruit and vegetable intake (% change)  Overall  Income quintiles 1 to 5  Similar impacts across income groups. | -  -  -  -  -  -  -  - | *Taxes regressive:* Effects were in the same direction across all income groups except for the best case scenario tax where lowest income group was estimated to consume less energy; all other income groups were estimated to consume more energy  *Compensation buying:* NR  *Definition of healthy/less healthy foods:* less healthy foods defined using SSCg3d [[40](#_ENREF_40)] nutrient profiling model  *Application and size of tax/subsidy:* Flat tax/subsidy on total cost applied at point of sale |
| Nordstrom (2007), Sweden [[26](#_ENREF_26),[27](#_ENREF_27)] | *Model:* Includes 8 major food categories: bakery goods, ready meals, flours and dough, soft bread, crisp bread, breakfast, pasta, and rice. | *Combinations:* 10.71% subsidy on healthier breads and cereals and 34.2% tax on bakery products and ready meals; 50% subsidy on healthier breads and cereals and 113.8% tax on bakery goods and ready meals; 0.046 subsidy per gram of fibre per kg of grain product and 0.182 tax per gram of added sugar; and 0.046 subsidy per gram of fibre per kg and 0.325 tax per gram of saturated fat | *Dataset:* GfK market research expenditure data combined with household expenditure data from Statistics Sweden  *N:* 1,192 and 1,104 respectively  *Socioeconomic group:* impact of interventions assessed across four income groups (1=lowest). | *10.7% bread and cereal subsidy and 34.2% bakery and ready meals tax:*  Bread and breakfast cereal purchases (% change):  Income groups 1 to 4  Bakery and ready meal purchases (% change):  Income groups 1 to 4  Nutrients - fibre, energy, salt, sugar, total fat, added sugar (% change):  Income groups 1 to 4  Saturated fat:  Income groups 1 to 4  *50% bread and cereal subsidy and 113.8% bakery and ready meals tax:*  Bread and breakfast cereal purchases (% change):  Income groups 1 to 4  Bakery and ready meal purchases (% change):  Income groups 1 to 4  Nutrients - fibre, saturated fat, energy, salt, sugar, total fat, added sugar (% change):  Income groups 1 to 4  *Fibre subsidy and grain tax:*  Bread and breakfast cereal purchases (% change):  Income groups 1 to 4  Bakery and ready meal purchases (% change):  Income groups 1 to 5  Nutrients (% change) - fibre, energy, salt, total fat Income groups 1 to 4  Added sugar:  Income groups 1 to 4  Saturated fat:  Income groups 1 & 2  Income groups 3 &4  Sugar:  Income group 1  Sugar income groups 2 to 4  *Fibre subsidy and saturated fat tax:*  Bread and breakfast cereal purchases (% change)  Income groups 1 to 5  Bakery and ready meal purchases (% change):  Income groups 1 to 5  Nutrients (% change)- fibre, energy, and salt  Income groups 1 to 4  Total fat:  Income groups 1 and 2  Income groups 3 and 4  Sugar, energy, and added sugar:  Income group 1  Income groups 2 to 4  Effect of combined taxes and subsidies appears even across income groups. | +  -  0^b^  +^b^  -  +  -  +  +  -  +  -  +  -  +  -  +  -  +  +  -  + | *Taxes regressive:* some nutrient tax and subsidy combinations may be regressive  *Compensation buying:* NR  *Definition of healthy/less healthy foods:* Using Swedish Keyhole nutrient criteria [[41](#_ENREF_41)]  *Application and size of tax/subsidy:* Combination of flat rate tax on total cost applied at point of sale and rate per gram of nutrient ( |
| Sassi (2009) U.S. [[30](#_ENREF_30)] | *Model:* Includes fruit and vegetables and total fat | *Combinations:* 10% tax on foods high in total fat and 10% subsidy on fruit and vegetables | *Dataset:* United Kingdom Family Food Survey 2007  *N:* NR  *Socioeconomic group:* Sensitivity analysis to assess effects across socioeconomic groups. | *Total fat tax and fruit and vegetable subsidy:*  Foods high in total fat  Sensitivity analysis indicated health effects tend to favour lower socioeconomic groups. | - | *Taxes regressive:* Yes  *Compensation buying:* NR  *Definition of healthy/less healthy foods:* NR  *Application and size of tax/subsidy:* Flat rate tax applied at point of sale |
| Smed (2007), Denmark [[32](#_ENREF_32)] | *Model:* Includes 23 food groups | *Combinations:* Tax on meat, butter and cheese and subsidy on fruit, vegetables, potatoes and grains (revenue neutral); saturated fat tax and fibre subsidy (revenue neutral);saturated fat and sugar tax and fibre subsidy (revenue neutral) | *Dataset:* Gfk consumer scan data (1997 to 2000)  *N:* ~2,000  *Socioeconomic group:* impact of combined interventions assessed across 5 social classes (1 = lowest). Varied impact on food purchases by social class. | *Saturated fat tax & fibre subsidy*  *Overall:*  Saturated fat  Sugar and fibre  *By social class:*  Saturated fat (class 1)  Saturated fat (classes 2 to 5)  Sugar (class 1)  Sugar (classes 2 to 5)  Fibre (classes 1 to 5)  *Saturated fat and sugar tax and fibre subsidy:*  *Overall:*  Saturated fat and sugar  Fibre  *By social class:*  Saturated fat (class 1)  Saturated fat (classes 2 to 5)  Fibre (classes 1 to 5)  Sugar (classes 1 & 2)  Sugar (classes 3 to 5) | -  +  +  -  -  +  +  -  +  +  -  +  +  - | *Taxes regressive:* Yes  *Compensation buying:* NR  *Definition of healthy/less healthy foods:* n/a  *Application and size of tax/subsidy:* Combination of flat taxes and rate per amount of nutrient applied at point of sale |
| Tiffin (2011), U.K. [[35](#_ENREF_35),[36](#_ENREF_36)] | *Model:* *Model:* Includes 7 food groups: milk; other dairy, eggs and fats; meat and fish; potatoes, rice, and pasta; cereals; fruit and vegetables; and drinks | *Combinations:* tax on ‘fatty’ foods of 1% per every % of saturated fat they contain (ceiling of 15%) and subsidy on fruit and vegetables to exactly cancel costs of saturated fat tax to consumers (~15%) | *Dataset:* U.K. Expenditure and Food Survey 2005 to 2006 (2-week diary for participants >7yrs)  *N:* NR  *Socioeconomic group:* NR | *Saturated fat tax and fruit and vegetable subsidy*  Nutrient purchases in % TE  Protein  Total fat  Saturated fat  Monounsaturated fat  Polyunsaturated fat  Sugar  (very small changes not clinically significant)  Nutrient purchases in absolute amounts  Energy (MJ)  Cholesterol (mg)  Sodium (cg)  Fibre (dg)  Fruit and vegetables (g)  (large change for fruit and vegetable purchases now in line with national recommendations) | 0  -  -  -  -  +  -  -  -  +  + | *Taxes regressive:* NR  *Compensation buying:* Effects assessed across range of nutrients  *Definition of healthy/less healthy foods:* n/a  *Application and size of tax/subsidy:* Combination of nutrient tax at 1% per % nutrient and subsidy at rate to = tax cost to consumer |

**Methods and findings of studies assessing the effects of pricing strategies on diet (food/ nutrient intake/purchases)**

| **First Author (year), Country** | **Model food groups** | **Intervention & Duration** | **Participants,**  **Groups & Setting** | **Outcomes** | **Impact** | **Quality** | **Pragmatic issues addressed** |
| --- | --- | --- | --- | --- | --- | --- | --- |
| **Impact on health and nutrition-related disease** | | | | | | | |
| **Ecological and simulation modelling studies** | | | | | | | |
| **Studies assessing taxes** | | | | | | | |
| Chaloupka (2011), U.S. (Illinois)[[8](#_ENREF_8)] | *Model:* Includes 2 types SSBs: all beverages and SSB only | *Taxes:*  One cent per ounce on SSBs; one cent per ounce on SSB and diet versions; two cent per ounce on SSB; and two cent per ounce on SSB and diet versions | *Dataset:* For beverages: Beverage Marketing Corporation sales (2009) and Beverage World sales (2009) extrapolated over period 2000 to 2009. For epidemiological model: 2009 Behavioural Risk Factor Surveillance System data for Illinois and 2007 survey of Children’s Health  *N:* NR but state sales  *Socioeconomic group:* NR | *One penny per ounce SSB:*  Diabetes incidence  Health care costs of diabetes  Obesity prevalence  Obesity-related health care costs  Tax revenues ($U.S.606.7 million)  *One penny per ounce SSB + diet:*  Diabetes incidence  Health care costs of diabetes  Obesity prevalence  Obesity-related health care costs  Tax revenues ($U.S. 876.1 million)  *Two penny per ounce SSB:*  Diabetes incidence  Health care costs of diabetes  Obesity prevalence  Obesity-related health care costs  Tax revenues ($U.S. 839.3 million)  *Two penny per ounce SSB + diet:*  Diabetes incidence  Health care costs of diabetes  Obesity prevalence  Obesity-related health care costs  Tax revenues ($U.S. 1,419.6 million) | -  -  -  -  -  -  -  -  -  -  -  -  -  -  -  - |  | *Taxes regressive:* n/a  *Compensation buying:* Estimated using literature to be 50% of energy intake to other beverages  *Definition of healthy/less healthy foods:* n/a  *Application and size of tax/subsidy:* Rate per volume applied at point of sale |
| Chouinard (2007), U.S.[[9](#_ENREF_9)] | *Model:* Includes 14 categories of dairy products | *Taxes:*  10% and 50% | *Dataset:* Info scanner data from 1997 to 1999 for 23 U.S. cities  *N:* ~50,000 to 10 million per city  *Socioeconomic group:* Burden of tax examined by household income. | *10% fat tax on dairy:*  Body weight  *50% fat tax on dairy:*  Body weight  Similar findings across income groups therefore reported overall only | 0  0 | *Own- and cross- PEs included:* Y  *Model validation:* N  *Sensitivity analyses:* N | *Taxes regressive:* elasticities similar across demographic groups, but burden of tax falls predominantly on lowest income groups  *Compensation buying:* NR  *Definition of healthy/less healthy foods:* n/a  *Application and size of tax/subsidy:* Flat tax on total cost applied at point of sale |
| Clarke (2010) U.K. [[10](#_ENREF_10)] | *Model:* Includes 18 major food categories | *Taxes:*  3%, 5%, 10% and 20% taxes on: all foods; less healthy and intermediate foods (with no tax on any fruit and vegetables); and less healthy foods | *Dataset:* Jersey Household Expenditure Survey 2005 combined with expenditure and consumption data from the United Kingdom Family Food report 2005  *N:* NR  *Socioeconomic group:* NR | *All food tax:*  Deaths averted or delayed from CHD, stroke, and diet-related cancers  All rates  (1 to 12 lives lost as tax increases)  *Less healthy and intermediate healthy food tax:*  All rates  (1 to 5 lives lost as tax increases)  *Less healthy tax:*  All rates  (0.5 to 3 lives lost as tax increases) | -  -  - | *Own- and cross- PEs included:* Y  *Model validation:* NR  *Sensitivity analyses:* Y | *Taxes regressive:* NR  *Compensation buying:* NR  *Definition of healthy/less healthy foods:* foods categorised into healthier, intermediate and less healthy using SSCg3d nutrient profiling model [[40](#_ENREF_40)]  *Application and size of tax/subsidy:* Flat rate tax applied at point of sale |
| Dharmasena (2011), U.S. [[11](#_ENREF_11)] | *Model:* Includes 10 categories of non-alcoholic beverages: sports drinks, regular and diet soft drinks, whole and low fat milk, fruit drinks and juices, bottled water, coffee and tea. | *Taxes:*  20% on sugar three categories of sweetened beverages: sports drinks, regular soft drinks, and fruit drinks | *Dataset:* Nielsen Homescan panel data for four regions in U.S. from 1998 to 2003  *N:* NR  *Socioeconomic group:* NR | *Selected SSB tax:*  Body weight  (~ 3lb or 1.6 kg / year) | - | *Own- and cross- PEs included:* Y  *Model validation:* Elasticities and findings compared in tables with similar studies  *Sensitivity analyses:* N | *Taxes regressive:* NR  *Compensation buying:* NR  *Definition of healthy/less healthy foods:* n/a  *Application and size of tax/subsidy:* Flat rate tax applied at point of sale |
| Fantuzzi (2008) U.S. [[13](#_ENREF_13)] | *Model:* Includes 26 brands of carbonated soft drinks | *Taxes:*  20% flat rate on soft drinks, and U.S. 10c per calorie rate on soft drinks | *Dataset:* Scanner data for 20 U.S. cities  *N:* 40,000  *Socioeconomic group:* effects assessed for lower and higher income groups. | *Flat rate soft drink tax:*  Low and high incomes:  Body weight  *Per nutrient soft drink tax:*  Body weight  (very small changes ~1lb (450g)/year)  Higher PEs and impact on lower income groups. | -  - | *Own- and cross- PEs included:* N  *Model validation:* NR  *Sensitivity analyses:* NR | *Taxes regressive:* Yes *Compensation buying:* NR  *Definition of healthy/less healthy foods:* n/a  *Application and size of tax/subsidy:* Combination of flat rate tax on total cost and tax amount per calorie applied at point of sale |
| Fletcher (2008), U.S. [[16](#_ENREF_16)] | *Model:* Two food categories: soft drinks and other foods | *Taxes:*  Variation in sales taxes across 50 states between 1990 and 2006 used to estimate mean tax increase of average 3% on soft drinks; two rates estimated based on total tax and incremental tax | *Dataset:* Behavioural Risk Factor Surveillance System national surveys 1990 to 2006  *N:* 2,709,422  *Socioeconomic group:* Effects by two ethnic groups explored: Hispanic and Black. | *Total soft drink tax:*  Overweight  Obesity  BMI  Black BMI  Hispanic BMI  *Incremental soft drink tax:*  Overweight  Obesity  BMI  Black BMI  Hispanic BMI  Effects on body weight and BMI greater for Whites and Blacks than Hispanic. | -  0  -  -  -  -  -  -  -  - | *Own- and cross- PEs included:* Y  *Model validation:* NR  *Sensitivity analyses:* Y | *Taxes regressive: NR*  *Compensation buying:* NR  *Definition of healthy/less healthy foods:* n/a  *Application and size of tax/subsidy:* Flat rate tax on total cost applied at point of sale (rates per volume were converted to flat rates) |
| Gelbach (2007) U.S. [[18](#_ENREF_18)] | *Model:* Three food types: healthful (apples, bananas, grapefruit, grapes, lemons, Navel oranges, Valencia oranges, peaches, tomatoes); unhealthful (bacon, ice cream, sugar); and all food | *Taxes:*  100% tax on bacon, ice cream and white sugar | *Dataset:* National Health Interview Survey data 1982 to 1996  *N:* NR  *Socioeconomic group:* analysis djusted for ethnicity, income and education | *Bacon, ice cream and white sugar tax:*  BMI  (very small changes ~1%) | - | *Own- and cross- PEs included:* N  *Model validation:* NR  *Sensitivity analyses:* NR | *Taxes regressive:* NR  *Compensation buying:* NR  *Definition of healthy/less healthy foods:* Healthy foods = 9 types of fruit including tomatoes and less healthy = bacon, ice cream and white sugar  *Application and size of tax/subsidy:* Flat rate tax on total cost applied at point of sale |
| Kotakorpi (2011) Finland [[39](#_ENREF_39)] | *Model:* Includes 6 food categories: bread, meat, fish, fruit and vegetables, sugar and sweets, and other | *Taxs:* 1€ per kg added sugar | *Dataset:* Finish Household Budget Surveys 1995, 1998, 2001, & 2006 for demand models and Health Survey 2000 for change in food intake and nutrients  *N:* Household budget surveys  ~17,000 households; Health Survey =10,000 individuals  *Socioeconomic group:* three income groups based on disposable income (low,med,high). | *Sugar tax:*  Body weight:  Low income  Middle income  High income  (-.8 to -5.4kg; Sig = p<0.05)  Incidence of type 2 diabetes:  Low income  Middle income  High income  (-3 to -21%; Sig=p<0.05)  Incidence of CHD:  Low income  Middle income  High income  Higher elasticities for low income groups therefore health impacts greater and overall inequalities decreased | - (Sig)  -  -  - (Sig)  -  -  - (Sig)  -  - | *Taxes regressive:* Yes, mildly.  *Compensation buying:* Yes, but with only potential effect detrimental to health being increased meat intake  *Application and size of tax/subsidy:* Rate per amount of nutrient applied at point of sale | *Definition of healthy/less healthy foods:* n/a  *Application and size of tax/subsidy:* Rate per amount of nutrient applied at point of sale |
| Kuchler (2005), U.S.  [[21](#_ENREF_21)] | *Model:* Includes 3 categories of snack foods: potato chips, all chips, and salty snacks | *Taxes:*  1%, 10% and 20% taxes on potato chips, all chips, and salty snacks | *Dataset:* AC Nielsen homescan panel sales data 1999  *N:* 12,000 households in panel for ≥ 10 months  *Socioeconomic group:* NR | *1%, 10% and 20% potato chip taxes:*  Weight loss  (very small changes; <1 pound)  *1%, 10% and 20% all chips taxes:*  Weight loss  (very small changes)  *1%, 10% and 20% all salty snacks taxes:*  Weight loss  (very small changes) | -  -  - | *Own- and cross- PEs included:* N (only across snack foods)  *Model validation:* N  *Sensitivity analyses:* N | *Taxes regressive:* NR  *Compensation buying:* NR  *Definition of healthy/less healthy foods:* n/a  *Application and size of tax/subsidy:* Flat tax on total cost applied at point of sale |
| Marshall (2000), U.K. [[23](#_ENREF_23)] | *Model:* Includes 6 categories of food contributing to saturated fat in British diet: whole milk, cheese, butter, biscuits, buns cakes and pastries, puddings and ice cream | *Taxes:*  17.5% on 6 categories of food contributing to saturated fat in British diets | *Dataset:* Dietary intake data only used from 1990 survey of British adults  *N:* NR  *Socioeconomic group:* NR | *17.5% taxes on foods contributing to saturated fat intakes:*  Serum cholesterol (absolute amount)  Total cholesterol (~-0.05mmol/L)  (small reductions all <0.1mmol/L)  Ischemic heart disease (%)  Total (~-2%)  Number of deaths avoided:  Total men  Total women | -  -  ~1,000  ~600 | *Own- and cross- PEs included:* N  *Model validation:* N  *Sensitivity analyses:* N | *Taxes regressive:* NR  *Compensation buying:* NR  *Definition of healthy/less healthy foods:* food contributing substantially to saturated fat intakes in Britain  *Application and size of tax/subsidy:* Flat tax on total cost applied at point of sale |
| Mytton (2007), U.K. [[24](#_ENREF_24)] | *Model:* Includes 18 major food categories | *Taxes:*  17.5% on principle sources of saturated fat; less healthy foods; and combination of taxes to achieve best health outcome for lowest cost to consumer | *Dataset:* National Food Survey of Great Britain 2000  *N:* NR  *Socioeconomic group:* NR | *Saturated fat tax:*  Serum cholesterol (mean change)  Mortality from ischemic heart disease (% change)  Mortality from stroke (% change)  Annual deaths from CVD (% change)  *Less healthy food tax:*  Serum cholesterol (mean change)  Mortality from ischemic heart disease (% change)  Mortality from stroke (% change)  Annual deaths from CVD (% change)  *Best outcome tax:*  Serum cholesterol (mean change)  Mortality from ischemic heart disease (% change)  Mortality from stroke (% change)  Annual deaths from CVD (% change) | +  +  +  +2,500 to 3,100  +  -  -  2,100 to 2,500  +  -  -  -2,600 to 3,200 | *Own- and cross- PEs included:* Y  *Model validation:* NR  *Sensitivity analyses:* Completed to check assumptions of estimated cross PEs | *Taxes regressive:* NR  *Compensation buying:* NR  *Definition of healthy/less healthy foods:* less healthy foods defined using SSCg3d [[40](#_ENREF_40)] nutrient profiling model  *Application and size of tax/subsidy:* Flat tax on total cost applied at point of sale |
| Nnoaham (2009), U.K. [[25](#_ENREF_25)] | *Model:* Includes ~18 major food categories | *Taxes:*  17.5% on principle sources of saturated fat; less healthy foods; and combination of taxes to achieve best health outcome for lowest cost to consumer  vegetables | *Dataset:* Expenditure and Food Survey 2003 to 2006  *N:* NR  *Socioeconomic group:* effects assessed across 5 categories of household income (1=lowest). | *Saturated fat tax^a^:*  Annual deaths CHD (change)  Annual deaths stroke (change)  Annual deaths cancer (change)  Annual deaths CVD (change)  *Less healthy food tax:*  Annual deaths CHD (change)  Annual deaths stroke (change)  Annual deaths cancer (change)  Annual deaths CVD (change)  *Best case scenario tax:*  Annual deaths CHD (change)  Annual deaths stroke (change)  Annual deaths cancer (change)  Annual deaths CVD (change)  Similar impacts across income groups. | ~ -112  ~+234  ~+199  ~ -8  ~ -119  ~ +118  ~+ 129  ~ -96  ~ -345  ~ -322  ~ -375  ~ +16 | *Own- and cross- PEs included:* Y  *Model validation:* NR  *Sensitivity analyses:* Completed to check assumptions of estimated cross PEs | *Taxes regressive:* Yes  *Compensation buying:* NR  *Definition of healthy/less healthy foods:* less healthy foods defined using SSCg3d [[40](#_ENREF_40)] nutrient profiling model  *Application and size of tax/subsidy:* Flat tax/subsidy on total cost applied at point of sale |
| Oaks (2005), U.S. [[28](#_ENREF_28)] | *Model:* ecological study where tax in Maine compared with no tax in New Hampshire. | *Taxes:*  Evaluation of soft drink and snack tax in Maine from 1991 to 2001 at rate of 5.5% on soft drinks, snack foods, carbonated water, ice cream, and toasted pastries | *Dataset:* Behavioural Risk Factor Surveillance System national surveys for four years prior to tax and 1991 to 2001  *N:* NR  *Socioeconomic group:* income included in regression model but effects by group not assessed | *Soft drink and snack food tax:*  BMI | 0 | *Own- and cross- PEs included:* Some *Model validation:* NR  *Sensitivity analyses:* NR | *Taxes regressive:* NR  *Compensation buying:* NR  *Definition of healthy/less healthy foods:* NR although specifically noted tax was confusing for consumers and retailers as inconsistencies in which items were taxes  *Application and size of tax/subsidy:* Flat rate tax on total cost applied at point of sale |
| Sacks (2010), Australia [[29](#_ENREF_29)] | *Model:* Includes 9 food categories: regular bread and rolls, cereal-based products and dishes, cheese, muscle meat, poultry and other feathered game, sausages frankfurters and saveloys, snack foods, confectionery, soft drinks flavoured mineral waters and electrolyte drinks | *Taxes:*  10% on ‘junk food’ | *Dataset:* National Nutrition Survey data 1995  *N:* NR  *Socioeconomic group:* NR | *Junk (less healthy) food tax:*  Weight loss for males and females (kg)  Overall population weight loss (kg)  (~ 1 to 2 kg in each case) | -  - | *Own- and cross- PEs included:* Y  *Model validation:* NR  *Sensitivity analyses:* NR | *Taxes regressive:* NR  *Compensation buying:* NR  *Definition of healthy/less healthy foods:* less healthy foods selected based on non-core foods high in saturated fat, sugar, and/or salt  *Application and size of tax/subsidy:* Flat rate tax applied at point of sale |
| Schroeter (2008), U.S.  [[31](#_ENREF_31)] | *Model:* Includes 2 categories of food: high calorie food, and low calorie food | *Taxes:*  10% on food away from home and regular soft drinks | *Dataset:* 2004 data on U.S. average daily per-capita consumption; body weight data from National Health and Examination Surveys 1963 to 1965 and 1999 to 2002; exercise data from 1965 and 2001 national time use surveys  *N:* NR  *Socioeconomic group:* NR | *Food away from home tax:*  Body weight (average kg increase)  Male  Female  (changes small <0.2 kg)  *Regular soft drink tax:*  Body weight (average kg increase)  Male  Female  (changes small <0.1 kg) | +  +  -  - | *Own- and cross- PEs included:* Y  *Model validation:* NR  *Sensitivity analyses:* NR | *Taxes regressive:* NR  *Compensation buying:* food away from home tax estimated to increase meat consumption  *Definition of healthy/less healthy foods:* n/a  *Application and size of tax/subsidy:* Flat tax/subsidy on total cost applied at point of sale |
| Smith (2010), U.S. [[33](#_ENREF_33)] | *Model:* Includes 8 categories of soft drinks based on energy content: caloric sweetened beverages, diet drinks, skim milk, low fat milk, 100% fruit and vege juices, coffee/tea, and bottled water | *Taxes:*  20% on caloric sweetened beverages | *Dataset:* Nielsen Homescan household scanner data 1998 to 2007 and National Health and Examination Survey data 2003 to 2006  *N:* NR  *Socioeconomic group:* NR | *Caloric sweetened beverage tax:*  Weight loss:  Adults  Children  Prevalence of overweight:  Adults  Children  (greater decline for children)  Prevalence of obesity:  Adults  Children  (greater decline for children) | -  -  -  -  -  - | *Own- and cross- PEs included:* only across different categories of beverage  *Model validation:* NR  *Sensitivity analyses:* NR | *Taxes regressive:* NR  *Compensation buying:* NR outside of beverages  *Definition of healthy/less healthy foods:* n/a  *Application and size of tax/subsidy:* Flat rate tax applied at point of sale |
| **Studies assessing subsidies** | | | | | | | |
| Cash (2005), U.S. [[7](#_ENREF_7)] | *Model:* Includes 3 categories of fruit and vegetables | *Subsidies:*  1% on fruit and vegetables | *Dataset:* US continuing study of food intakes (1994 to 1996 and 1998)  *N:* 18,081 >2yrs of age  *Socioeconomic group:* Impact on number of lives saved by disease examined across 3 income groups | *1% subsidy providing a lasting price reduction in all fruit and vegetables*  Number of cases of CHD prevented:  All incomes  Low income  Medium income  High income  Number of cases of Ischemic stroke disease prevented:  All incomes  Low income  Medium income  High income  Fewer lives saved for lower compared with middle and high income. | 6,903  1,152  2,260  3,492  3,022  568  997  1,457 | *Own- and cross- PEs included:* N  *Model validation:* N  *Sensitivity analyses:*  Monte Carlo analyses included. | *Taxes regressive:* n/a  *Compensation buying:* NR  *Definition of healthy/less healthy foods:* n/a  *Application and size of tax/subsidy:* Flat tax on total cost applied at point of sale |
| Kotakorpi (2011) Finland [[39](#_ENREF_39)] | *Model:* Includes 6 food categories: bread, meat, fish, fruit and vegetables, sugar and sweets, and other | *Subsidies:* 13% VAT removal on fresh fruit and vegetables and fish | *Dataset:* Finish Household Budget Surveys 1995, 1998, 2001, & 2006 for demand models and Health Survey 2000 for change in food intake and nutrients  *N:* Household budget surveys  ~17,000 households; Health Survey =10,000 individuals  *Socioeconomic group:* three income groups based on disposable income (low,med,high). | *Fresh fruit and vegetable and fish subsidy:*  Incidence of CHD:  All income groups  Higher elasticities for low income groups therefore health impacts greater and overall inequalities decreased | Mixed findings as relative risks not added together difficult to determine overall impact | *Taxes regressive:* Yes, mildly.  *Compensation buying:* Yes, but with only potential effect detrimental to health being increased meat intake  *Application and size of tax/subsidy:* Rate per amount of nutrient applied at point of sale | *Definition of healthy/less healthy foods:* n/a  *Application and size of tax/subsidy:* Flat subsidy applied at point of sale |
| Schroeter (2008), U.S.  [[31](#_ENREF_31)] | *Model:* Includes 2 categories of food: high calorie food, and low calorie food | *Subsidies:*  10% on fruit and vegetables and diet soft drinks | *Dataset:* 2004 data on U.S. average daily per-capita consumption; body weight data from National Health and Examination Surveys 1963 to 1965 and 1999 to 2002; exercise data from 1965 and 2001 national time use surveys  *N:* NR  *Socioeconomic group:* NR | *Fruit and vegetable subsidy:*  Body weight (average kg increase)  Male  Female  (changes small <0.2 kg)  *Diet soft drink subsidy:*  Body weight (average kg increase)  Male  Female  (changes small <0.1 kg) | +  +  -  - | *Own- and cross- PEs included:* Y  *Model validation:* NR  *Sensitivity analyses:* NR | *Taxes regressive:* n/a  *Compensation buying:* food away from home tax estimated to increase meat consumption  *Definition of healthy/less healthy foods:* n/a  *Application and size of tax/subsidy:* Flat tax/subsidy on total cost applied at point of sale |
| **Studies assessing combinations of taxes and subsidies** | | | | | | | |
| Clarke (2010) U.K. [[10](#_ENREF_10)] | *Model:* Includes 18 major food categories | *Combinations:* 3%, 5%, 10% and 20% taxes on less healthy foods and corresponding equal subsidies on fruit and vegetables; and 3%, 5%, 10% and 20% taxes on less healthy foods and revenue neutral subsidies on fruit and vegetables | *Dataset:* Jersey Household Expenditure Survey 2005 combined with expenditure and consumption data from the United Kingdom Family Food report 2005  *N:* NR  *Socioeconomic group:* NR | *Less healthy tax and fruit and vegetable subsidy*  Number of lives saved:  All rates  (1 to 5 lives saved as rate increases)  *Less healthy tax and fruit and vegetable subsidy (revenue neutral)*  Number of lives saved  All rates  (2 to 19 lives saved as rate increases) | +  + | *Own- and cross- PEs included:* Y  *Model validation:* NR  *Sensitivity analyses:* Y | *Taxes regressive:* NR  *Compensation buying:* NR  *Definition of healthy/less healthy foods:* foods categorised into healthier, intermediate and less healthy using SSCg3d nutrient profiling model [[40](#_ENREF_40)]  *Application and size of tax/subsidy:* Flat rate tax applied at point of sale |
| Kotakorpi (2011) Finland [[39](#_ENREF_39)] | *Model:* Includes 6 food categories: bread, meat, fish, fruit and vegetables, sugar and sweets, and other | *Combinations:* 1€ per kg of added sugar tax and 13% VAT removal on fresh fruit and vegetables and fish | *Dataset:* Finish Household Budget Surveys 1995, 1998, 2001, & 2006 for demand models and Health Survey 2000 for change in food intake and nutrients  *N:* Household budget surveys  ~17,000 households; Health Survey =10,000 individuals  *Socioeconomic group:* three income groups based on disposable income (low,med,high). | *Sugar tax and fresh fruit and vegetable and fish subsidy:*  Body weight:  All income groups  (-2.3kg average; Sig=p<0.05)  Incidence of type 2 diabetes:  All income groups  (9.7% average: Sig=p<0.05)  Incidence of CHD:  All income groups  (Sig not reported)  Higher elasticities for low income groups therefore health impacts greater and overall inequalities decreased | - (Sig)  -(Sig)  - | *Taxes regressive:* Yes, mildly.  *Compensation buying:* Yes, but with only potential effect detrimental to health being increased meat intake  *Application and size of tax/subsidy:* Rate per amount of nutrient applied at point of sale | *Definition of healthy/less healthy foods:* n/a  *Application and size of tax/subsidy:* Flat subsidy applied at point of sale |
| Nnoaham (2009), U.K. [[25](#_ENREF_25)] | *Model:* Includes ~18 major food categories | *Combinations:* 17.5% tax on less healthy foods combined with subsidy on fruit and vegetables | *Dataset:* Expenditure and Food Survey 2003 to 2006  *N:* NR  *Socioeconomic group:* effects assessed across 5 categories of household income (1=lowest). | *Tax + subsidy:*  Annual deaths CHD (change)  Annual deaths stroke (change)  Annual deaths cancer (change)  Annual deaths CVD (change)  Similar impacts across income groups. | ~ -234  ~ -103  ~ -133  ~ -48 | *Own- and cross- PEs included:* Y  *Model validation:* NR  *Sensitivity analyses:* Completed to check assumptions of estimated cross PEs | *Taxes regressive:* Change in annual deaths in same direction across all 5 income groups^a^  *Compensation buying:* NR  *Definition of healthy/less healthy foods:* less healthy foods defined using SSCg3d [[40](#_ENREF_40)] nutrient profiling model  *Application and size of tax/subsidy:* Flat tax/subsidy on total cost applied at point of sale |
| Tiffin (2011), U.K. [[35](#_ENREF_35),[36](#_ENREF_36)] | *Model:* Includes 7 food groups: milk; other dairy, eggs and fats; meat and fish; potatoes, rice, and pasta; cereals; fruit and vegetables; and drinks | *Combinations:* tax on ‘fatty’ foods of 1% per every % of saturated fat they contain (ceiling of 15%) and subsidy on fruit and vegetables to exactly cancel costs of saturated fat tax to consumers (~15%) | *Dataset:* U.K. Expenditure and Food Survey 2005 to 2006 (2-week diary for participants >7yrs)  *N:* NR  *Socioeconomic group:* NR | *Saturated fat tax and fruit and vegetable subsidy (RR)*  Gastric cancer  Lung cancer  CVD  CHD  All chronic disease  Ischemic stroke | -  -  -  -  -  - | *Own- and cross- PEs included:* Y  *Model validation:* NR  *Sensitivity analyses:* NR | *Taxes regressive:* NR  *Compensation buying:* Effects assessed across range of nutrients  *Definition of healthy/less healthy foods:* n/a  *Application and size of tax/subsidy:* Combination of nutrient tax at 1% per % nutrient and subsidy at rate to = tax cost to consumer |

**Key to Appendix:**

U.S., United States; U.K., United Kingdom

SSB: Sugar-sweetened beverage

TE, Total energy; MJ, mega joule (1,000 kJ); g, gram; mg, milligram

0, no difference between groups/no change in purchases; + increase in purchases/consumption; -, decrease in purchases/consumption;

NR, not reported or not estimated; n/a, not applicable

CHD, Coronary Heart Disease; CVD, Cardiovascular disease; BMI, Body Mass Index

^a^ too many data to be reported by socioeconomic group

^b^ very small differences (<0.1%) may have been present between socioeconomic groups, although data summarised for this table

**References**

1. Ulubasoglu M, Mallick D, Wadud M, Hone P, Haszler H (2010) Food demand elasticities in Australia. Accessed 3rd February 2012. Available at: <http://ideas.repec.org/p/dkn/econwp/eco_2010_17.html>. Melbourne: Deakin University

2. Ministry of Agriculture Fisheries and Food (2000) National food survey 2000: Annual report on food expenditure, consumption and nutrient intakes. Her Majesty's Stationery Office.

3. Main C, Thomas S, Ogilvie D, Stirk L, Petticrew M, et al. (2008) Population tobacco control interventions and their effects on social inequalities in smoking: placing an equity lens on existing systematic reviews. BMC Public Health 8: doi: 10.1186/1471-2458-1188-1178.

4. Allais O, Bertail P, Nichele V (2010) The effects of a fat tax on French households' purchases: a nutritional approach. Amer J Agr Econ 92: 228-244.

5. Andreyeva T, Chaloupka FJ, Brownell KD (2011) Estimating the potential of taxes on sugar-sweetened beverages to reduce consumption and generate revenue. Prev Med 52: 413-416.

6. Bahl R, Bird R, Walker MB (2003) The uneasy case against discriminatory excise taxation: soft drink taxes in Ireland. Public Financ Rev 31: doi:10.1177.1091142103253753.

7. Cash HH, Davis DE, LaFrance JT, Perloff JM (2005) Fat taxes and thin subsidies: prices, diet, and health outcomes. Acta Agr Scand 2: 167-174.

8. Chaloupka FJ, Wang YC, Powell LM, Andreyeva T, Chriqui JF, et al. (2011) Estimating the potential impact of sugar-sweetened and other beverage excise taxes in Illinois. Accessed 25th January 2011. Available at: <http://www.cookcountypublichealth.org/files/pdf/Chaloupka_Report_PRF.pdf>. Illinois: Cook County Department of Public Health

9. Chouinard HH, Davis DE, LaFrance JT, Perloff JM (2007) Fat taxes: big money for small change. Forum for Health Economics and Policy 10.

10. Clarke D, Scarborough P, Rayner M (2010) Estimating the effects of different food tax and subsidy scenarios on the health of the population of Jersey (unpublished). London: University of Oxford

11. Dharmasena S, Capps O (2011) Intended and unintended consequences of a proposed national tax on sugar-sweetended beverages to combat the U.S. obesity problem. Health Econom: doi:10.1002/hec.1738.

12. Dong D, Lin B-H (2009) Fruit and vegetable consumption by low-income Americans. Accessed 25th January 2012. Available at: <http://www.ers.usda.gov/publications/err70/err70.pdf>. United States Department of Agriculture

13. Fantuzzi K (2008) Carbonated soft drink consumption: implications for obesity policy. Accessed 25th January 2012. Available at: <http://digitalcommons.uconn.edu/dissertations/AAI3313272/:> University of Connecticut.

14. Lopez AD, Fantuzzi K (2010) Carbonated soft drink choices and obesity. Connecticut: University of Connecticut

15. Finkelstein EA, Zhen C, Nonnemaker J, Todd JE (2010) Impact of targeted beverage taxes on higher- and lower-income households. Arch Intern Med 170: 2028-2034.

16. Fletcher JM, Tefft N (2010) Can soft drink taxes reduce population weight? Cont Econ Policy 28: 23-35.

17. Gabe T (2008) Fiscal and economic impacts of beverage excise taxes imposed by Maine public law 629. Maine: University of Maine

18. Gelbach JB, Kilick J, Stratmann T (2007) Cheap donuts and expensive broccoli: the effect of relative prices on obesity. Accessed 25th January 2012. Available at: <http://papers.ssrn.com/sol3/papers.cfm?abstract_id=976484>. Tallahassee: Florida State University College of Law

19. Gustavsen G (2005) Public policies and the demand for carbonated soft drinks: a censored quantile regression approach. European Association of Agricultural Economists. Copenhagen.

20. Jensen JD, Smed S (2007) Cost-effective design of economic instruments in nutrition policy. Int J Behav Nutr Phys Activity 4: doi:10.1186/1479-5868-1184-1110.

21. Kuchler F, Tegene A, Harris JM (2005) Taxing snack foods: manipulating diet quality or financing information programs? Rev Ag Eco 27: 4-20.

22. LaCroix A, Muller L, Ruffieux B (2010) To what extent would the poorest consumers nutritionally and socially benefit from a global food tax and subsidy reform? A framed field experiment based on daily food intake. Accessed 25th January 2012. Available at: <http://ideas.repec.org/p/gbl/wpaper/201004.html>. Universite Pierre Mendes France

23. Marshall T (2000) Exploring a fiscal food policy: the case of diet and ischemic heart disease. Brit Med J 320: 301-305.

24. Mytton O, Gray A, Rayner M, Rutter H (2007) Could targeted food taxes improve health? J Epidemiol Community Health 61: 689-694.

25. Nnoaham KE, Sacks G, Rayner M, Mytton O, Gray A (2009) Modelling income group differences in the health and economic impacts of targeted food taxes and subsidies. Int J Epidemiol 38: 1324-1333.

26. Nordstrom J, Thunstrom L (2009) The impact of tax reforms designed to encourage healthier grain consumption. J Health Econ 28: 622-634.

27. Nordstrom J, Thunstrom L (2010) Can targeted food taxes and subsidies improve the diet? Distributional effects among income groups Food Policy 36: 259-271.

28. Oaks B (2005) An evaluation of the snack tax on the obesity rate of Maine. Accessed 25th January 2012. Available at: <http://ecommons.txstate.edu/cgi/viewcontent.cgi?article=1029&context=arp&sei-redir=1&referer=http%3A%2F%2Fwww.google.co.nz%2Furl%3Fsa%3Dt%26rct%3Dj%26q%3Doaks%2Bsnack%2Btax%26source%3Dweb%26cd%3D1%26ved%3D0CCwQFjAA%26url%3Dhttp%253A%252F%252Fecommons.txstate.edu%252Fcgi%252Fviewcontent.cgi%253Farticle%253D1029%2526context%253Darp%26ei%3DBGMfT9-HCOeQiAefwanVDQ%26usg%3DAFQjCNEH3NE8MhCIn7y-daPR45Ml6BKg1A#search=%22oaks%20snack%20tax%22>. Maine: Texas State University

29. Sacks G, Veerman JL, Moodie M, Swinburn B (2010) 'Traffic-light' nutrition labelling and 'junk-food' tax: a modelled comparison of cost-effectiveness for obesity prevention. Int J Obesity 35: 1001-1009.

30. Sassi F, Cecchini M, Lauer J, Chisholm D (2009) Improving lifestyles, tackling obesity: the health and economic impact of prevention strategies. Accessed 25th January 2012. Available at: <http://www.oecd-ilibrary.org/docserver/download/fulltext/5ks5pqlc5jnn.pdf?expires=1327450398&id=id&accname=guest&checksum=3F4E5794A347D00869DF3E02B68E7D5B>. Geneva: World Health Organization

31. Schroeter C, Lusk J, Tyner W (2008) Determining the impact of food price and income changes on body weight. J Health Econ 27: 45-68.

32. Smed S, Jensen J, Denver S (2007) Socio-economic characteristics and the effect of taxation as a healthy policy instrument. Food Policy 32: 624-639.

33. Smith TA, Lin B-H, Lee J-Y (2010) Taxing caloric sweetened beverages: potential effects on beverage consumption, calorie intake and obesity. Accessed 25th January 2011. Available at: <http://www.ers.usda.gov/Publications/err100/err100.pdf>. U.S. Department of Agriculture

34. Tefft N (2008) The effects of a soft drink tax on household expenditures. Accessed 28th January 2012. Available at: <http://abacus.bates.edu/~ntefft/research/soft_drink_taxes_ces.pdf>. Bates College

35. Tiffin R, Arnoult M (2011) The public health impacts of a fat tax. Eur J Clin Nutr 65: 427-433.

36. Arnoult MH, Tiffin R, Traill WB (2008) Models of nutrient demand, tax policy and public health impact. Reading: University of Reading

37. Zhen C, Wohlgenant MK, Karns S, Kaufman P (2011) Habit formation and demand for sugar-sweetened beverages. Am J Ag Econ 93: 175-193.

38. Johnson SR, Hassan ZA, Green RD (1984) Demand system estimation: methods and applications: Iowa State University Press.

39. Kotakorpi K, Harkanen T, Pietinen P, Reinivuo H, Suoniemi I, et al. (2011) The welfare effects of health-based food tax policy. Accessed 25th January 2012. Available at: <http://papers.ssrn.com/sol3/papers.cfm?abstract_id=1959273>. CESinfo Working Paper No. 3633

40. Rayner M, Scarborough P, Stockley L (2004) Nutrient profiles: options for definitions for use in relation to food promotion and children's diets. London: Food Standards Agency

41. Lobstein T, Davies S (2009) Defining and labellilng 'healthy' and 'unhealthy' food. Public Health Nutr 12: 331-340.
